# Relocatable modular capacities in risk aware strategic supply network planning under demand uncertainty

**DOI:** 10.1007/s41471-022-00141-z

**Published:** 2022-11-03

**Authors:** Ariane Kayser, Florian Sahling

**Affiliations:** 1 Department of Production Management, Leibniz Universität Hannover, Königsworther Platz 1, 30167 Hannover, Germany; 2 Chair of Production Management, University of Kaiserslautern, Gottlieb-Daimler-Straße, 67663 Kaiserslautern, Germany

**Keywords:** Supply network reconfiguration, Modular capacities, Stochastic demand, Conditional value at risk

## Abstract

We propose a new model formulation for a three-echelon supply network design problem incorporating the concept of relocatable modular capacities. A robust supply network configuration must be determined based on uncertain demand. Furthermore, by incorporating the conditional value at risk (CVaR), the risk induced by uncertain demand is explicitly considered. The derived supply network configuration should maximize the weighted sum of the expected net present value and the CVaR. The resulting nonlinear model formulation is approximated by a piecewise linearization. Our numerical investigation shows that the derived supply network configuration is robust and stable in the presence of uncertain demand.

## Introduction

The location of production facilities, the selection of vendors, and the assignment of retailers to production facilities are strategic decisions often faced by managers. Due to changes in a company’s environment, supply networks must be dynamically reconfigurable. Such changes might include changes in demand or cost structures over time. For example, to meet a geographical shift in demand, companies might want to move their production facilities from regions with decreasing demand to regions with increasing demand to reduce transportation expenses.

To enable the rapid relocation of production facilities, the concept of modular capacities arises. According to this concept, production facilities consist of freely combinable modules of different types that can easily be relocated. This enables the relocation of individual modules or even the entire production facility. The ability to relocate modular capacities and reuse them at different locations is an important aspect when considering the ecological effects of (re)designing supply networks, as the waste of raw materials and resources can be reduced by relocating and reusing these modules. In addition, while the modules rarely have to be relocated, numerous regular transports of the products to the retailers over long distances can be avoided.

As an example, relocatable modular capacities will be used for the production of the mRNA-based COVID-19 vaccine in Africa; see BioNTech (2022). The capacity of each production facility can be expanded by additional modules. In this way, further production sites can easily be added to the production network of the COVID-19 vaccine. A further example is the final assembly of cars; see Bala (2014). Different module types can be used to form a complete modular production line and therefore establish flexible production facilities. The modules can easily be stowed in containers for transportation purposes. If demand increases, modules can be added at the site to establish an additional production line, whereas if demand decreases, production capacity can be reduced by removing lines (i.e., by removing/relocating the respective modules).

**Fig. 1:**
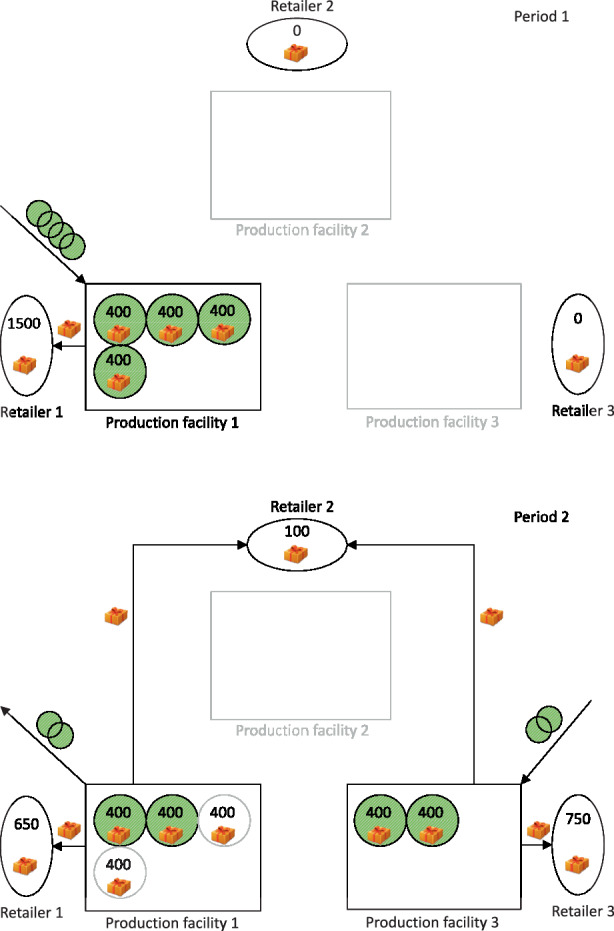
Configuration of a supply network with deterministic demand without relocation of modular capacities

**Fig. 2:**
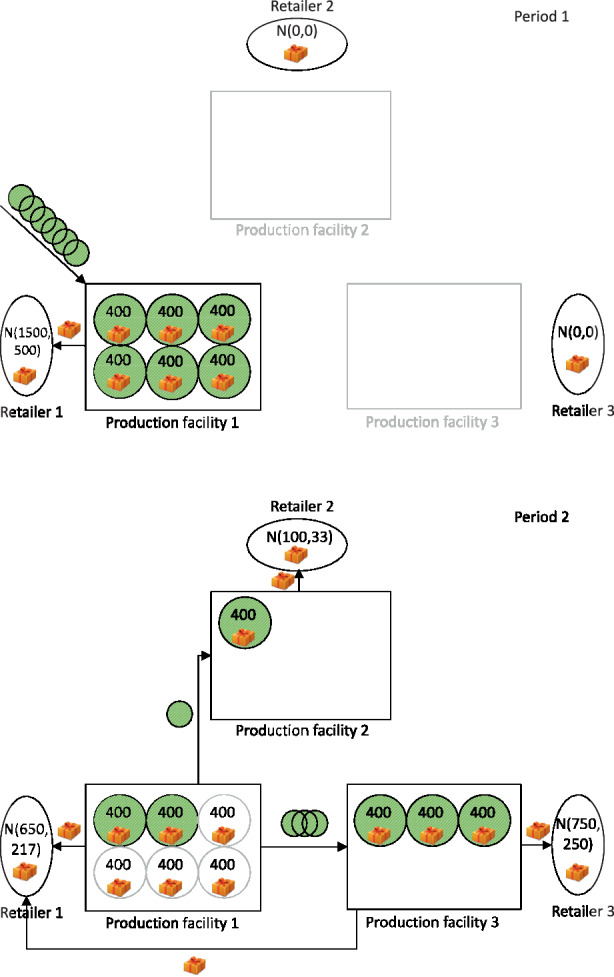
Configuration of a supply network with stochastic demand with relocation of modular capacities

Because of the long-term planning horizon related to strategic decisions, uncertainty must be considered when decisions regarding the supply network structure are made. One source of uncertainty is future demand. Thus, to sustain profitability, a supply network must be configured that is robust to changes in future demand. Figs. 1 and 2 present a comparative example that illustrates the concept of (relocatable) modular capacities in the presence of shifting (un)certain demand.

In Fig. 1, the demand is assumed to be deterministic, and relocating modules is not possible. Due to the shift in demand in the second period, modules from production facility 1 are sold, and an equivalent number of modules is purchased for the newly established production facility 3. However, in the case of stochastic demand, as shown in Fig. 2, a completely different supply network structure appears when the relocation of modules is enabled. Due to the demand’s uncertainty, more modules are acquired during the first period. During the second period, modules are relocated from the existing production facility 1 to the newly established production facilities 2 and 3. The resulting supply network differs considerably from the network described in Fig. 1.

To develop an effective supply network, decisions regarding the location of production facilities, the selection of vendors, and the allocation of retailers must be made simultaneously. The effectiveness of a supply network depends on the relations between the production and distribution echelons and, importantly, on the acquisition echelon of the network. For example, considerable distances between vendors and production facilities can have a significant impact on payments for acquiring components. Thus, a three-echelon supply network planning problem is presented in this paper. Furthermore, the production facilities consist of relocatable modules; therefore, the supply network can be reconfigured according to the acquisition, allocation, and relocation of modules.

Because of the long-term planning horizon, it is necessary to consider the net present value (NPV) of discounted cash flows. Furthermore, because risk is induced by uncertain demand, risk is explicitly taken into account by incorporating the conditional value at risk (CVaR). Thus, the weighted sum of the expected NPV of discounted cash flows and the CVaR is maximized in the objective function.

The consideration of uncertain demand leads to a nonlinear model formulation for supply network planning. Thus, following Helber et al. (2013), piecewise linear functions are used to approximate the stochastic nonlinear model formulation. A small number of linearization segments already allows for an adequate approximation without a substantial increase in numerical effort. A two-stage stochastic programming approach is applied and allows decisions to be revisited after a given period of time.

The remainder of this paper is structured as follows. In Sect. 2, we present a literature overview of strategic supply network planning in the presence of adaptable capacities. In Sect. 3, the stochastic network design problem with modular capacities underlying our model is presented. The model assumptions are stated in Sect. 3.1, and the piecewise approximation method is described in Sect. 3.2. In Sect. 4, our stochastic programming approach is presented. The linearized model formulation for the robust supply network design problem with modular capacities is stated in Sect. 4.1, and the two-stage stochastic programming approach is described in Sect. 4.2. In Sect. 5, we report the results of our numerical investigation. This paper ends with a summary and outlook in Sect. 6.

## Literature review

To respond to dynamic changes in a company’s environment, supply networks must be adjustable. This section presents an overview of papers that allow a supply network to be reconfigured by adjusting the facilities’ capacities and/or relocating capacities. An overview of these papers is presented in Table 1.

**Table 1:** Literature overview

	Number of echelons	Uncertainty	Capacity constraints				Facilities			Service level	Risk	Salvage value	
Paper			E	R	M	RL	O	C	RO				Objective
Aghezzaf (2005)	3	$\bullet$	$\bullet$										Min disc. costs
Allman and Zhang (2020)	2		$\bullet$	$\bullet$	$\bullet$	$\bullet$	$\bullet$	$\bullet$	$\bullet$				Min costs
Akbari et al. (2018)	2	$\bullet$			$\bullet$		$\bullet$						Min weight. sum of primary & backup coverage, mean access time dev. from goal
Antunes and Peeters (2001)	2		$\bullet$	$\bullet$	$\bullet$		$\bullet$	$\bullet$					Min disc. costs
Becker et al. (2019)	2		$\bullet$	$\bullet$	$\bullet$	$\bullet$	$\bullet$	$\bullet$	$\bullet$				Min costs
Behmardi and Lee (2008)	2		$\bullet$	$\bullet$		$\bullet$	$\bullet$	$\bullet$					Max NPV
Hammami and Frein (2014)	$\geq$3		$\bullet$	$\bullet$	$\bullet$	$\bullet$	$\bullet$	$\bullet$					Max profit
Jena et al. (2015a, b, 2016, 2017)	2		$\bullet$	$\bullet$	$\bullet$	$\bullet$	$\bullet$	$\bullet$	$\bullet$				Min costs
Lee (1991)	2				$\bullet$		$\bullet$						Min costs
Lee and Luss (1987)	2		$\bullet$			$\bullet$							Min costs
Martel (2005)	3		$\bullet$	$\bullet$	$\bullet$		$\bullet$	$\bullet$					Max after tax net revenues
Melachrinoudis and Min (2000)	3		($\bullet$)	($\bullet$)		$\bullet$	$\bullet$	$\bullet$					Max disc. profit, Min access time, Max local incentives
Melo et al. (2006, 2012, 2014)	$\geq$3		$\bullet$	$\bullet$	$\bullet$	$\bullet$	$\bullet$	$\bullet$					Min discounted costs
Paquet et al. (2004)	3		$\bullet$	$\bullet$	$\bullet$		$\bullet$	$\bullet$					Min costs
Shulman (1991)	2		$\bullet$	$\bullet$	$\bullet$							$\bullet$	Min disc. costs
Thanh et al. (2008)	3		$\bullet$		$\bullet$		$\bullet$	$\bullet$					Min costs
Troncoso and Garrido (2005)	2		$\bullet$		$\bullet$		$\bullet$						Min disc. costs
Vila et al. (2006)	3		$\bullet$	$\bullet$	$\bullet$		$\bullet$	$\bullet$					Max profit
Wilhelm et al. (2013)	$\geq$3		$\bullet$	$\bullet$	$\bullet$		$\bullet$	$\bullet$					Min disc. costs
**Our model**	**3**	$\bf\bullet$	$\bf\bullet$	$\bf\bullet$	$\bf\bullet$	$\bf\bullet$	$\bf\bullet$	$\bf\bullet$	$\bf\bullet$	$\bf\bullet$	$\bf\bullet$	$\bf\bullet$	**Max weighted sum of NPV and CVaR**

*Capacity constraints* Consideration of capacities ($E$ Capacity expansion, $M$ Modular capacities, $R$ Capacity reduction, *RL* Capacity relocation)

*Facilities* Consideration of facility status ($C$ Closing, $O$ Opening, *RO* Reopening)

*Objective* Objective function (*CVaR* Conditional Value at Risk, NPV Net Present Value)

The mathematical models in the stated papers vary widely regarding the integration of capacities and the possibility of changing them. We therefore focus on this characteristic in the following and present some of the methods used in more detail. Due to the single-period nature of the work of Lee (1991), capacity expansion, reduction, and relocation are not considered. The author does, however, consider modular capacities by incorporating the selection of a subset of possible facility types at a facility site. Facility types differ based on their specified capacity for the production of a particular product and their setup costs. Shulman (1991) presented a facility location problem that considers modular capacities to allow discrete expansion and reduction in the sizes of the considered plants. He defines a plant as a collection of facilities at the same location. The facilities differ by their capacities to produce the only considered product and by their installation costs. The number of facilities of a particular type at each location is limited. Antunes and Peeters (2001) introduced a dynamic model for modular capacitated school network planning. In their model, decisions are made regarding the opening of new schools and expanding, reducing, or closing existing schools. The facility sizes can be expanded or reduced according to a set of predefined standards (i.e., modules with a given capacity). A deterministic model formulation for a single-period network design problem is presented by Paquet et al. (2004). They introduce capacity options that can be implemented at a facility. These capacity options differ by capacity, required space, and their associated fixed and variable costs in the facilities. Troncoso and Garrido (2005) allow for capacity expansion at a site according to predefined levels. The number of expansions along the planning horizon is restricted. Martel (2005) presents a network design problem that incorporates capacity options for the design of a facility layout. In this paper, Martel (2005) assumes that a facility consists of a fixed part and a variable part. This variable part of the facility layout provides an area that can be used to install a number of capacity options. Capacity options differ by capacity, the required floor space, fixed costs and variable costs per product. The use of these capacity options enables the expansion or reduction of a facility. Vila et al. (2006) also use capacity options in their model to enable facility expansion and reduction in the lumber industry. In their model, the plant’s capacity depends on the chosen capacity option, which can be seasonally shut down. Melo et al. (2006) consider a model in which capacities are assumed to be modular. The module types differ by size and relocation costs. Thanh et al. (2008) consider the possibility of enlarging the manufacturer’s facilities. To enable this enlargement, decision makers can choose to add predefined capacity options. The capacity options vary by production and storage capacity, as well as fixed costs for opening and operating an option. In Wilhelm et al. (2013), capacity is modeled modularly, and various capacity alternatives are introduced. Each alternative is associated with a certain number of capacity modules, and capacity alternatives are defined as an integer multiple of the capacity of a basic module. A model that focuses on the redesign of an existing supply chain is presented by Hammami and Frein (2014), who consider capacity expansion/reduction, as well as capacity relocation. To achieve this purpose, the authors decompose the production process into different activities that come along with certain capacity requirements (e.g., a certain number of machines or production lines) that can be acquired either from an external source or relocated. Activities differ by acquisition, operation and relocation costs, production capacity and portfolio. A model formulation to find the optimal number, location and size of logging camps to accommodate changes in harvest areas is presented by Jena et al. (2015). In their work, the facilities are camps composed of trailers. Camp capacity can be expanded by adding trailers or reduced by closing trailers. In a more general version of this problem, Jena et al. (2015b) use capacity levels to implement modular capacities. Based on this modeling, Jena et al. (2016, 2017) focus on solving the dynamic facility location problem. The authors Silva et al. (2021) also develop heuristics for the model presented by Jena et al. (2015b). Becker et al. (2019) consider modular capacities for volume and process flexibility. The authors work with a set of production module configurations consisting of different process modules that differ by acquisition and installation costs as well as operating costs. Costs also occur when modules are reconfigured from one configuration to another. A similar approach is used by Allman and Zhang (2020). In their work, configurations are chosen for facilities. These configurations determine which modules are necessary for the production of one unspecified product. Modules differ by acquisition, setup and relocation costs. Relocation costs also depend on origin and destination.

The contribution of our paper is as follows: In our model, we divide capacity into modules of different module types. Module types differ not only by size and production capacity and acquisition, holding, relocation and production costs but also by production portfolio. Each module type can be used to produce a number of specified different products with a capacity consumption factor that also depends on the module type.

Furthermore, stochastic information is rarely considered in model formulations for the reconfiguration of supply networks, as indicated in Table 1. However, because of the long-term nature of strategic decisions regarding the reconfiguration of the supply network, the available information is typically characterized by high uncertainty at the time decisions must be made. Thus, decisions based on deterministic values can turn out to be very unfavorable due to information changes in the future. It is therefore advisable to explicitly consider the uncertainty of information in models regarding the supply network configuration. In our model, we consider two different forms of uncertainty for the demand. First, a period’s demand is considered to be a normally distributed random variable with an expected mean value and standard deviation. Second, we consider the estimate of the mean value of the random demand to be uncertain, i.e., the mean value for each period of the planning horizon may be either lower or higher (recession or boom). Thus, the uncertain demand is modeled as a mixture of normal distributions. This second form of uncertainty is especially important when entering a new market. Aghezzaf (2005) consider uncertain demand through the commonly used sample average approximation by using specific demand scenarios containing realizations of the random demand and their probability of occurrence. Akbari et al. (2018) use a goal programming technique to consider multiple objectives for locating maritime search and rescue vessel stations and allocating modular vessels. An aspiration level is chosen for each of the three objectives and the deviation considered in three respective restrictions under the presence of uncertain demand, i.e., occurrence of an emergency event. Uncertain demand is captured by a set of randomly generated demand scenarios, and each objective is calculated as the weighted average over all scenarios. The authors then minimize the weighted sum over all three objectives, i.e., all deviations. However, a significant number of scenarios is required to accurately describe the normal distribution of our random demand, which increases the numerical effort.

Hence, we use a piecewise linear approximation to incorporate the first form of uncertainty induced by the normally distributed random demand. This approximation allows a leaner model compared to the sample average approach. For the second form of uncertainty, we consider future scenarios, e.g., scenarios with overall low, normal and high expected mean values of demand. However, our future scenarios do not contain demand realizations. Instead, within each scenario, the demand is described by the aforementioned normally distributed random variable reflecting the first form of uncertainty. This requires the piecewise linear approximation method within each future scenario. Additionally, to further address the uncertainty, we present a two-stage stochastic programming approach that enables a recourse, i.e., decisions are made to be revisited after a given period of time. Although some of the papers’ proposals penalize shortfalls to a customer in the objective function through penalty costs, we ensure demand satisfaction by incorporating a service-level constraint.

Although costs or profit are often considered in the objective function despite the long-term planning horizon in the literature, we believe that such problems require consideration of the NPV, i.e., discounted incoming and outgoing payments. This NPV should also include the salvage value of modules and facilities at the end of the planning horizon. Additionally, when facing an uncertain environment, it is important for the decision maker to estimate the risk regarding the reliability of his objective. Because all but one of the proposed models are deterministic, none of the cited authors explicitly account for the risk induced by the uncertainty of information. Thus, we consider the risk induced by the second form of uncertain demand by including the CVaR in our objective function to avoid substantial negative deviations from the expected NPV. This enables the decision maker to further evaluate the generated solution and adjust the structure according to his risk propensity.

## A stochastic supply network design problem with relocatable modular capacities

### Model Assumptions

The aim of the robust supply network design problem with modular capacities (RSNDPMC) is to configure a three-echelon supply network according to a number of model assumptions, divided into the following categories:

#### Objective and risk awareness

Several future scenarios $(s\in\mathcal{S})$ for the demand are defined in advance, each with an estimated occurrence probability $\pi_{s}$. Each future scenario describes a possible development of random demand. Despite the introduction of future scenarios, the scenarios do not contain demand realizations, but the demand within a scenario is represented by a normally distributed random variable, described later in this chapter. The configuration of the supply network should maximize the weighted sum of the expected NPV of payments and the CVaR (according to Rockafellar and Uryasev (2002)). The NPV is discounted by the internal interest rate $i^{ \textit{wacc}}_{t}$ in period $t$ derived from the weighted average cost of capital (WACC).

#### Selection of production facilities

When potential production facility $f\in\mathcal{F}$ is established (i.e., opened for the first time) in period $t$, i.e., $\varphi^{ \textit{est}}_{ \textit{ft}}=1$, a payment $\textit{pay}^{ \textit{est}}_{ \textit{ft}}$ arises. In each period in which an established production facility $f$ is open, i.e., $\varphi^{ \textit{open}}_{ \textit{ft}}=1$, a payment $\textit{pay}^{ \textit{open}}_{ \textit{ft}}$ occurs. This payment occurs in each period as long as facility $f$ remains available for production – even if no production occurs during a respective period $t$. A payment $\textit{pay}^{ \textit{close}}_{ \textit{ft}}$ occurs when facility $f$ is closed at the beginning of period $t$, i.e., $\varphi^{ \textit{close}}_{ \textit{ft}}=1$. However, a facility can be reestablished after having been closed in a previous period.

#### Installation of modular capacities at production facilities

Each production facility $f$ must be equipped with a suitable production system. The production system considered is decomposable into several types of modules $(m\in\mathcal{M})$. Module types differ by production portfolio. A module type subset $\mathcal{M}_{p}$ can be used to produce end product $p\in\mathcal{P}$. An end product subset $\mathcal{P}_{m}$ can be produced on a module of type $m$. The acquisition and installation of one module of type $m$ at production facility $f$ in period $t$ leads to a payment $\textit{pay}^{ \textit{Macqu}}_{ \textit{mft}}$. The number of acquired modules of type $m$ at production facility $f$ at the beginning of period $t$ is given by the integer variable $N^{ \textit{Macqu}}_{ \textit{mft}}$. $N^{ \textit{Mhold}}_{ \textit{mft}}$ describes the number of modules of type $m$ held at production facility $f$ at the end of period $t$. A payment $\textit{pay}^{ \textit{Mhold}}_{ \textit{mft}}$ is induced per module. These payments include the cost for maintaining the modules up to a certain standard in each period. Each module of type $m$ has a space requirement of $\textit{sp}_{m}$. A production facility $f$ with a maximum available space of $\textit{SP}^{ \textit{max}}_{ \textit{ft}}$ in period $t$ consists of a number of freely combinable modules. The number of modules of type $m$ relocated from production facility $f$ and installed at production facility $f^{\prime}$ at the beginning of period $t$ is given by $N^{ \textit{Mreloc}}_{ \textit{mff't}}$. The relocation process leads to a payment $\textit{pay}^{ \textit{Mreloc}}_{ \textit{mff't}}$ per relocated module. Due to their regular maintenance, modules can be sold at an unspecified aftermarket for a period-independent incoming payment $\textit{pay}^{ \textit{Msell}}_{ \textit{mf}}$; however, $\textit{pay}^{ \textit{Msell}}_{ \textit{mf}}\leq \textit{pay}^{ \textit{Macqu}}_{ \textit{mft}}$ for each period $t$. Thus, trading modules are not profitable. $N^{ \textit{Msell}}_{ \textit{mft}}$ provides the number of modules of type $m$ sold from production facility $f$ at the beginning of period $t$.

#### Production process

The production quantity of end product $p$ on any module of type $m$ at facility $f$ is given by $q^{ \textit{Pprod}}_{ \textit{pmfrts}}$ for retailer $r$ in period $t$ and future scenario $s$. The production of one unit of end product $p$ on any module of type $m$ at production facility $f$ in period $t$ induces a payment $\textit{pay}^{ \textit{Pprod}}_{ \textit{pmft}}$. The production of one unit of end product $p$ on any module of type $m$ at facility $f$ in period $t$ requires a capacity consumption $\textit{cf}^{ \textit{Pprod}}_{ \textit{pmft}}$. The production capacity of one module of type $m$ is restricted by $\textit{cap}^{ \textit{Mmax}}_{ \textit{m}}$ in each period. The production capacity $\textit{cap}^{ \textit{Mmax}}_{ \textit{m}}$ of a module of type $m$ at production facility $f$ in period $t$ is also affected by the installation of a newly acquired module, $\textit{cf}^{ \textit{Macqu}}_{ \textit{mft}}$, or the relocation of a module, $\textit{cf}^{ \textit{Mreloc}}_{ \textit{mf'ft}}$. $\textit{cf}^{ \textit{Mreloc}}_{ \textit{mf'ft}}$ includes the installation and the capacity loss due to transportation lag. The relocation capacity consumption is accounted for at the receiving facility.

#### Vendor selection to procure components

For the production of one unit of end product $p$, $u_{ \textit{cp}}$ units of component $c\in\mathcal{C}$, offered by vendors $v\in\mathcal{V}_{c}\subseteq\mathcal{V}$, are needed. The subset $\mathcal{P}_{c}$ contains those end products that require component $c$. The procurement of component $c$ from vendor $v$ in period $t$ induces a payment $\textit{pay}^{ \textit{Cacqu}}_{ \textit{cvt}}$ per unit. The transportation quantities are indicated by $q^{ \textit{Ctrans}}_{ \textit{cvfts}}$. A payment $\textit{pay}^{ \textit{Ctrans}}_{ \textit{cvft}}$ arises for transporting one unit of component $c$ from vendor $v$ to facility $f$ in period $t$. For each future scenario $s$ and each period $t$ in which an order of component $c$ is placed at vendor $v$, i.e., $\vartheta^{ \textit{Corder}}_{ \textit{cvts}}=1$, a payment $\textit{pay}^{ \textit{Corder}}_{ \textit{cvt}}$ occurs. The procurement quantity of component $c$ from vendor $v$ must be between the minimum $\textit{cap}^{ \textit{Cmin}}_{ \textit{cv}}$ and the maximum $\textit{cap}^{ \textit{Cmax}}_{ \textit{cv}}$ if the respective component is procured during the actual period.

#### Consideration of stochastic demand

At retailer $r$, end product $p$ can be sold according to the demand for an incoming payment $\textit{pay}^{ \textit{Psell}}_{ \textit{prt}}$ per unit in period $t$. Transporting one unit of end product $p$ from facility $f$ to retailer $r$ in period $t$ yields a payment $\textit{pay}^{ \textit{Ptrans}}_{ \textit{pfrt}}$. The random variable $\mathbf{D}_{ \textit{prts}}$ describes the demand for end product $p$ at retailer $r\in\mathcal{R}$ in period $t\in\mathcal{T}$ for each future scenario $s$. It is assumed that $\mathbf{D}_{ \textit{prts}}$ is normally distributed and the expected value ${\mathbb{E}}[\mathbf{D}_{ \textit{prts}}]$ and variance $\text{VAR}[\mathbf{D}_{ \textit{prts}}]$ are known. Furthermore, the random variables $\mathbf{D}_{ \textit{prts}}$ are pairwise stochastically independent. If the demand $\mathbf{D}_{ \textit{prts}}$ for end product $p$ at retailer $r$ exceeds the cumulated quantity of products produced in period $t$ and future scenario $s$, lost sales $\mathbf{LS}_{ \textit{prts}}$ may occur, i.e.,

1 $$\mathbf{LS}_{ \textit{prts}}=\max\left\{0,\mathbf{D}_{ \textit{prts}}-\sum_{m\in\mathcal{M}_{p}}\sum_{f\in\mathcal{F}}q_{ \textit{pmfrts}}^{ \textit{Pprod}}\right\}.$$

Expected lost sales ${\mathbb{E}}[\mathbf{LS}]$ can be derived by the *first-order-loss function*. The following explanations relate to a normally distributed demand $\mathbf{D}\sim\mathcal{N}(\mu_{D},\sigma_{D})$. For a given (cumulative) production quantity $q$, the expected standardized lost sales $\text{I\,E}[\widetilde{\mathbf{LS}}]$ for the standardized (cumulative) production quantity $v=\frac{q-\mu_{D}}{\sigma_{D}}$ corresponds to the value of the nonlinear first-order loss function $\Phi^{1}(v)$, which is defined as

2 $$\Phi^{1}(v)=\int_{ \textit{v}}^{\infty}(x-v)\cdot\phi(x)\cdot d\,x=\phi(v)-v\cdot\{1-\Phi(v)\},$$

where $\Phi(x)$ is the cumulated distribution function of a standardized normally distributed random variable $\mathbf{X}\sim\mathcal{N}(0,1)$ with $\mathbf{X}=\frac{\mathbf{D}-\mu_{D}}{\sigma_{D}}$. We refer to Tempelmeier (2020) for a more detailed description.

Furthermore, a $\beta$-service level is incorporated to ensure the satisfaction of the predetermined portion $\beta_{ \textit{pr}}$ of expected demand ${\mathbb{E}}[\mathbf{D}_{ \textit{prts}}]$.

### Approximation via piecewise linear functions

**Fig. 3:**
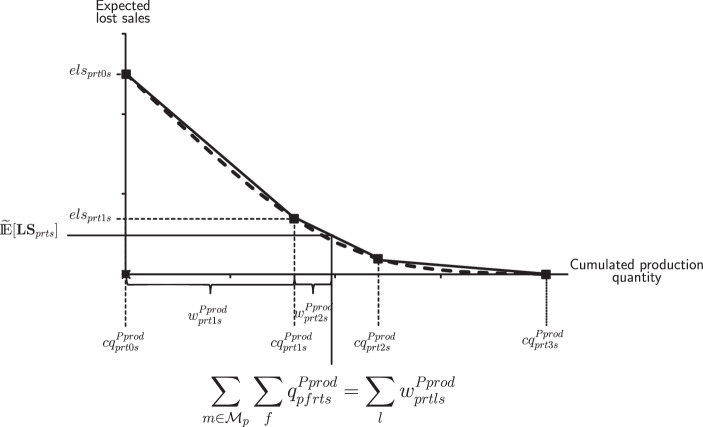
Linearization of the expected lost sales function, according to Sahling and Kayser (2016)

To address the nonlinearity of the expected lost sales, we suggest an approximation approach based on piecewise linear functions. This results in a mixed-integer linear model formulation that can be solved by any standard solver. Following Helber et al. (2013), the nonlinear function of expected lost sales is replaced by a piecewise linear function. Based on the chosen number of segments, this nonlinear function can be approximated with arbitrary precision. The linearization of the expected lost sales is illustrated in Fig. 3.

The expected lost sales ${\mathbb{E}}[\mathbf{LS}_{ \textit{prts}}]$ can be linearized by defining $L$ segments $(l=0,\ldots,L)$. The upper limit of each segment corresponds to supporting points $( \textit{els}_{ \textit{prtls}},\,cq^{ \textit{Pprod}}_{ \textit{prtls}})$ where the expected lost sales $\textit{els}_{ \textit{prtls}}$ can be calculated by the first-order-loss function (2) for a given cumulated production quantity $\textit{cq}^{ \textit{Pprod}}_{ \textit{prtls}}$. The first supporting point is required at the point of zero cumulative production, and the last supporting point is required at the point of maximum possible cumulative production. In accordance with Helber et al. (2013), the remaining $L-1$ supporting points are defined around the expected value of demand, i.e., in the area of the strongest nonlinearity.

Thus, the expected lost sales ${\mathbb{E}}[\mathbf{LS}_{ \textit{prts}}]$ can be approximated as follows:

3 $$\begin{aligned}{\mathbb{E}}[\mathbf{LS}_{ \textit{prts}}]\approx\widetilde{{\mathbb{E}}}[\mathbf{LS}_{ \textit{prts}}]= \textit{els}_{ \textit{prt0s}}+\sum_{l\in\mathcal{L}}\Delta^{ \textit{LS}}_{ \textit{prtls}}\cdot w^{ \textit{Pprod}}_{ \textit{prtls}}\quad\qquad\quad\qquad\forall p\in\mathcal{P},r\in\mathcal{R},t\in\mathcal{T},s\in\mathcal{S},\end{aligned}$$

where the slope $\Delta^{ \textit{LS}}_{ \textit{prtls}}$ of the linearized function is defined as

$$\Delta^{ \textit{LS}}_{ \textit{prtls}}=\frac{ \textit{els}_{ \textit{prtls}}- \textit{els}_{ \textit{prt,l-1,s}}}{cq^{ \textit{Pprod}}_{ \textit{prtls}}-cq^{ \textit{Pprod}}_{ \textit{prt,l-1,s}}}.$$

The parameter $\textit{els}_{ \textit{prt0s}}$ describes the expected lost sales in the case of no cumulated production. Furthermore, the additional decision variable $w^{ \textit{Pprod}}_{ \textit{prtls}}$ represents the portion of the cumulated production quantity related to linearization segment $l$.

Note that due to the direction of optimization and the convexity of the nonlinear function of expected lost sales proved by Rossi et al. (2014), this approximation approach ensures that the variables $w^{ \textit{Pprod}}_{ \textit{prtls}}$ are correctly determined, i.e., $w^{ \textit{Pprod}}_{ \textit{prtls}}$ take values starting at the first segment.

## A programming approach for the RSNDPMC

### A linear model formulation of the RSNDPMC

Based on the presented assumptions, the piecewise linear approximation of the RSNDPMC, RSNDPMC-PLA, can be stated using the notation in 1:

#### Objective function

In the objective function (4), the weighted sum of the expected NPV of discounted cash flows and the CVaR is maximized.

4 $$\begin{aligned}\max Z=\psi\cdot\sum_{s\in\mathcal{S}}\pi_{s}\cdot\text{NPV}_{s}+(1-\psi)\cdot\text{CVaR}\end{aligned}$$

The decision maker’s risk propensity is described by the parameter $\psi\in[0,1]$. In the case of a highly risk-aware decision maker $(\psi=0)$, only the CVaR is maximized. With an increasing $\psi$, which may take any value in the interval $]0,1]$, the risk awareness decreases, and only the expected NPV is maximized if $\psi=1$.

#### NPV-related constraints

The future-scenario-specific expected net present value $\text{NPV}_{s}$ of discounted cash flows is defined in (4a) to (4i).

4a $$\begin{aligned}\text{NPV}_{s}=\sum_{t\in\mathcal{T}}\frac{1}{\prod_{\tau=1}^{t}\left(1+i^{ \textit{wacc}}_{\tau}\right)}\cdot\left(\sum_{p\in\mathcal{P}}\sum_{r\in\mathcal{R}} \textit{pay}^{ \textit{Psell}}_{ \textit{prt}}\cdot\left({\mathbb{E}}[\mathbf{D}_{ \textit{prts}}]-\widetilde{{\mathbb{E}}}[\mathbf{LS}_{ \textit{prts}}]\right)\right.\end{aligned}$$

4b $$\begin{aligned}\quad\qquad+\sum_{m\in\mathcal{M}}\sum_{f\in\mathcal{F}} \textit{pay}^{ \textit{Msell}}_{ \textit{mf}}\cdot N^{ \textit{Msell}}_{ \textit{mft}}\end{aligned}$$

4c $$\begin{aligned}\quad\qquad-\sum_{m\in\mathcal{M}}\sum_{f\in\mathcal{F}}\Big( \textit{pay}^{ \textit{Macqu}}_{ \textit{mft}}\cdot N^{ \textit{Macqu}}_{ \textit{mft}}+ \textit{pay}^{ \textit{Mhold}}_{ \textit{mft}}\cdot N^{ \textit{Mhold}}_{ \textit{mft}}+\sum_{f^{\prime}\in\mathcal{F}\setminus f} \textit{pay}^{ \textit{Mreloc}}_{ \textit{mff't}}\cdot N^{ \textit{Mreloc}}_{ \textit{mff't}}\Big)\end{aligned}$$

4d $$\begin{aligned}\quad\qquad-\sum_{f\in\mathcal{F}}\Big( \textit{pay}^{ \textit{est}}_{ \textit{ft}}\cdot\varphi^{ \textit{est}}_{ \textit{ft}}+ \textit{pay}^{ \textit{open}}_{ \textit{ft}}\cdot\varphi^{ \textit{open}}_{ \textit{ft}}+ \textit{pay}^{ \textit{close}}_{ \textit{ft}}\cdot\varphi^{ \textit{close}}_{ \textit{ft}}\Big)\end{aligned}$$

4e $$\begin{aligned}\quad\qquad-\sum_{p\in\mathcal{P}}\sum_{m\in\mathcal{M}_{p}}\sum_{f\in\mathcal{F}}\sum_{r\in\mathcal{R}} \textit{pay}^{ \textit{Pprod}}_{ \textit{pmft}}\cdot q^{ \textit{Pprod}}_{ \textit{pmfrts}}\end{aligned}$$

4f $$\begin{aligned}\quad\qquad-\sum_{p\in\mathcal{P}}\sum_{m\in\mathcal{M}_{p}}\sum_{f\in\mathcal{F}}\sum_{r\in\mathcal{R}} \textit{pay}^{ \textit{Ptrans}}_{ \textit{pfrt}}\cdot q^{ \textit{Pprod}}_{ \textit{pmfrts}}\end{aligned}$$

4g $$\begin{aligned}\quad\qquad-\sum_{c\in\mathcal{C}}\sum_{v\in\mathcal{V}_{c}} \textit{pay}^{ \textit{Corder}}_{ \textit{cvt}}\cdot\vartheta^{ \textit{Corder}}_{ \textit{cvts}}\end{aligned}$$

4h $$\begin{aligned}\quad\qquad-\left.\sum_{c\in\mathcal{C}}\sum_{v\in\mathcal{V}_{c}}\sum_{f\in\mathcal{F}}\Big( \textit{pay}^{ \textit{Cacqu}}_{ \textit{cvt}}+ \textit{pay}^{ \textit{Ctrans}}_{ \textit{cvft}}\Big)\cdot q^{ \textit{Ctrans}}_{ \textit{cvfts}}\right)\end{aligned}$$

4i $$\begin{aligned}\quad\qquad+\frac{1}{\prod_{\tau=1}^{ \textit{T+1}}\left(1+i^{ \textit{wacc}}_{\tau}\right)}\cdot\left(\sum_{m\in\mathcal{M}}\sum_{f\in\mathcal{F}}\frac{ \textit{pay}^{ \textit{Macqu}}_{ \textit{mf,T+1}}+ \textit{pay}^{ \textit{Msell}}_{ \textit{pf}}}{2}\cdot N^{ \textit{Mhold}}_{ \textit{mfT}}\right.\left.+\sum_{f\in\mathcal{F}}\frac{ \textit{pay}^{ \textit{est}}_{ \textit{f,T+1}}- \textit{pay}^{ \textit{close}}_{ \textit{f,T+1}}}{2}\cdot\varphi^{ \textit{open}}_{ \textit{fT}}\right)\end{aligned}$$

$$\quad\qquad\qquad\forall s\in\mathcal{S}$$

The first part of (4a) to (4b) includes the expected incoming payments. Eq. (4a) represents the incoming payments for selling end products at the retailers, whereas (4b) represents incoming payments due to the selling of modules. The second part (4c) to (4h) contains all the remaining (outgoing) payments made by the company. In (4c), payments are considered for the acquisition, holding and relocation of modules. The term (4d) incorporates the payments for establishing, running and closing production facilities. (4e) contains payments for producing end products, whereas (4f) represents payments for the transportation of end products from the facilities to the retailers. The terms (4g) and (4h) are payments regarding the acquisition of components from vendors. In (4g), payments are considered for ordering components. The term (4h) incorporates payments for acquiring and transporting components. The last part of the objective function, (4i), represents the salvage value of the modules and the salvage value of the production facilities owned by the company at the end of the planning horizon.

For simplification purposes, the salvage values are calculated as the unweighted average of two events. First, the module in hold turns out to be unnecessary in the first period $T+1$ after the end of the planning horizon and would then be sold. Thus, it is worth its selling price $\textit{pay}^{ \textit{Msell}}_{ \textit{m,f}}$. Second, if this module would not be in hold in period $T+1$ and had to be acquired instead, the module is worth $\textit{pay}^{ \textit{MAcqu}}_{ \textit{m,f,T+1}}$. The same applies for the salvage value of the facilities. Here, the payments for closing $- \textit{pay}^{ \textit{close}}_{ \textit{f,T+1}}$ and establishing $\textit{pay}^{ \textit{est}}_{ \textit{f,T+1}}$ must be taken into account.

#### CVaR-related constraints

For a given probability $\alpha\in(0,1)$, the unbounded decision variable CVaR gives the mean value of the expected NPV of those future scenarios, whose NPV belongs to the worst $(1-\alpha)\cdot 100\%$ scenario-specific $\text{NPV}_{s}$. Following Fábián (2008) and Koberstein et al. (2013), ${\lvert}\mathcal{S}{\rvert}+1$ auxiliary decision variables are introduced for the calculation of the CVaR. The unbounded auxiliary decision variable $\omega_{0}$ gives a threshold value. For future scenarios whose expected NPV lies below the value of $\omega_{0}$, the ${\lvert}\mathcal{S}{\rvert}$ positive auxiliary decision variables $\omega_{s}$ take the value of the difference between $\omega_{0}$ and the respective future scenario-specific expected NPV. The CVaR is incorporated through constraints (5) and (6).

5 $$\begin{aligned}\text{CVaR}=\omega_{0}-\frac{1}{1-\alpha}\cdot\sum_{s\in\mathcal{S}}\pi_{s}\cdot\omega_{s}\quad\qquad\end{aligned}$$

6 $$\begin{aligned}\omega_{0}-\omega_{s}\leq\text{NPV}_{s}\quad\qquad\forall s\in\mathcal{S}\end{aligned}$$

In combination with the objective function (4), the constraints (5) and (6) ensure that the CVaR indeed gives the average of the worst $(1-\alpha)\cdot 100\%$ scenario-specific $\text{NPV}_{s}$.

#### Demand fulfillment

7 $$\begin{aligned}\widetilde{{\mathbb{E}}}[\mathbf{LS}_{ \textit{prts}}]= \textit{els}_{ \textit{prt0s}}+\sum_{l\in\mathcal{L}}\Delta^{ \textit{LS}}_{ \textit{prtls}}\cdot w^{ \textit{Pprod}}_{ \textit{prtls}}\quad\qquad\forall p\in\mathcal{P},r\in\mathcal{R},t\in\mathcal{T},s\in\mathcal{S}\end{aligned}$$

8 $$\begin{aligned}\sum_{s\in\mathcal{S}}\pi_{s}\cdot\widetilde{{\mathbb{E}}}[\mathbf{LS}_{ \textit{prts}}]\leq(1-\beta_{ \textit{pr}})\cdot\sum_{s\in\mathcal{S}}\pi_{s}\cdot{\mathbb{E}}[\mathbf{D}_{ \textit{prts}}]\quad\qquad\forall p\in\mathcal{P},r\in\mathcal{R},t\in\mathcal{T}\end{aligned}$$

9 $$\begin{aligned}\sum_{m\in\mathcal{M}_{p}}\sum_{f\in\mathcal{F}}q^{ \textit{Pprod}}_{ \textit{pmfrts}}=\sum_{l\in\mathcal{L}}w^{ \textit{Pprod}}_{ \textit{prtls}}\quad\qquad\forall p\in\mathcal{P},r\in\mathcal{R},t\in\mathcal{T},s\in\mathcal{S}\end{aligned}$$

10 $$\begin{aligned}w^{ \textit{Pprod}}_{ \textit{prtls}}\leq cq^{ \textit{Pprod}}_{ \textit{prtls}}-cq^{ \textit{Pprod}}_{ \textit{prt,l-1,s}}\quad\qquad\forall p\in\mathcal{P},r\in\mathcal{R},t\in\mathcal{T},l\in\mathcal{L},s\in\mathcal{S}\end{aligned}$$

Equalities (7) yield the approximated lost sales function presented in Sect. 3.2. Using inequalities (8), lost sales are restricted by a target $\beta$-service level. According to equations (9), the cumulated production quantity over all facilities must equal the cumulated production quantity of all linearization segments. The cumulated production $w^{ \textit{Pprod}}_{ \textit{prtls}}$ must not exceed the difference of the upper and lower bounds related to segment $l$; see (10).

#### Vendor-related constraints

11 $$\begin{aligned}\sum_{v\in\mathcal{V}_{c}}q^{ \textit{Ctrans}}_{ \textit{cvfts}}=\sum_{p\in\mathcal{P}_{c}}\sum_{m\in\mathcal{M}_{p}}\sum_{r\in\mathcal{R}}u_{ \textit{cp}}\cdot q^{ \textit{Pprod}}_{ \textit{pmfrts}}\quad\qquad\forall c\in\mathcal{C},f\in\mathcal{F},t\in\mathcal{T},s\in\mathcal{S}\end{aligned}$$

12 $$\begin{aligned}\sum_{f\in\mathcal{F}}q^{ \textit{Ctrans}}_{ \textit{cvfts}}\geq \textit{cap}^{ \textit{Cmin}}_{ \textit{cv}}\cdot\vartheta^{ \textit{Corder}}_{ \textit{cvts}}\quad\qquad\forall c\in\mathcal{C},v\in\mathcal{V}_{c},t\in\mathcal{T},s\in\mathcal{S}\end{aligned}$$

13 $$\begin{aligned}\sum_{f\in\mathcal{F}}q^{ \textit{Ctrans}}_{ \textit{cvfts}}\leq \textit{cap}^{ \textit{Cmax}}_{ \textit{cv}}\cdot\vartheta^{ \textit{Corder}}_{ \textit{cvts}}\quad\qquad\forall c\in\mathcal{C},v\in\mathcal{V}_{c},t\in\mathcal{T},s\in\mathcal{S}\end{aligned}$$

Eqs. (11) ensure that a sufficient amount of component $c$ is acquired to manufacture end products. Furthermore, the constraints (12) and (13) are the minimum and maximum capacity constraints for the components at vendors.

#### Constraints regarding modular capacities

Eqs. (14) to (16) consider the modular capacities in the model.

14 $$\begin{aligned}N^{ \textit{Mhold}}_{ \textit{mf,t-1}}+N^{ \textit{Macqu}}_{ \textit{mft}}+\sum_{f^{\prime}\in\mathcal{F}\setminus\{f\}}N^{ \textit{Mreloc}}_{ \textit{mf'ft}}-\sum_{f^{\prime}\in\mathcal{F}\setminus\{f\}}N^{ \textit{Mreloc}}_{ \textit{mff't}}-N^{ \textit{Msell}}_{ \textit{mft}}=N^{ \textit{Mhold}}_{ \textit{mft}}\quad\qquad\forall m\in\mathcal{M},f\in\mathcal{F},t\in\mathcal{T}\end{aligned}$$

15 $$\begin{aligned}\sum_{m\in\mathcal{M}}N^{ \textit{Mhold}}_{ \textit{mft}}\cdot \textit{sp}_{m}\leq \textit{SP}^{ \textit{max}}_{ \textit{ft}}\cdot\varphi^{ \textit{open}}_{ \textit{ft}}\quad\qquad\forall f\in\mathcal{F},t\in\mathcal{T}\end{aligned}$$

16 $$\begin{aligned}cf^{ \textit{Macqu}}_{ \textit{mft}}\cdot N^{ \textit{Maqcu}}_{ \textit{mft}}+\sum_{p\in\mathcal{P}_{m}}\sum_{r\in\mathcal{R}}cf^{ \textit{Pprod}}_{ \textit{pmft}}\cdot q^{ \textit{Pprod}}_{ \textit{pmfrts}}+\sum_{f^{\prime}\in\mathcal{F}\setminus\{f\}}cf^{ \textit{Mreloc}}_{ \textit{mf'ft}}\cdot N^{ \textit{Mreloc}}_{ \textit{mf'ft}}\quad\quad\qquad\leq \textit{cap}^{ \textit{Mmax}}_{m}\cdot N^{ \textit{Mhold}}_{ \textit{mft}}\quad\forall m\in\mathcal{M},f\in\mathcal{F},t\in\mathcal{T},s\in\mathcal{S}\end{aligned}$$

The balance constraints (14) determine the number of modules held at a facility in a period, considering the acquisition, relocation and selling of modules. Eqs. (15) ensure that the required space of the modules held at a production facility does not exceed its available space if the facility is open. Eqs. (16) represent the capacity consumption. Capacity consumption due to the acquisition of new modules, the relocation of modules, and the production of end products must not exceed the available production capacity according to the number of modules held at the respective production facility. Therefore, a facility must be equipped with modules to allow for production.

#### Selection of production facilities

17 $$\begin{aligned}\varphi^{ \textit{open}}_{ \textit{ft}}=\varphi^{ \textit{open}}_{ \textit{f,t-1}}+\varphi^{ \textit{est}}_{ \textit{ft}}-\varphi^{ \textit{close}}_{ \textit{ft}}\quad\qquad\forall f\in\mathcal{F},t\in\mathcal{T}\end{aligned}$$

Eqs. (17) ensure that production facility $f$ can operate only $(\varphi^{ \textit{open}}_{ \textit{ft}}=1)$ in period $t$ if the facility was established in the respective period $(\varphi^{ \textit{est}}_{ \textit{ft}}=1)$ or if it was open during the previous period $(\varphi^{ \textit{open}}_{ \textit{f,t-1}}=1)$. Payments for an open facility do not depend on the number of modules, i.e., payments may occur even if no modules are assigned (see (4d)). After a facility has been shut down, it can be reestablished in a later period. In combination with equations (15), modules must be removed from a facility in the case of a shut down. Furthermore, our approach can also be used to reconfigure a given supply network configuration, i.e., $\varphi^{ \textit{open}}_{ \textit{f0}}=1$ for some $f\in\mathcal{F}$.

### A two-stage stochastic programming approach

The sole solution of the RSNDPMC-PLA leads to a robust supply network configuration over all future scenarios. However, it is realistic to assume that the arising future scenario becomes apparent in the course of the ongoing planning horizon. Thus, the remodifications of the supply network configuration may become reasonable. Therefore, a two-stage stochastic programming approach is proposed that enables this future scenario-specific modification.

In the first stage, the complete RSNDPMC-PLA is solved to generate a robust supply network configuration that maximizes the weighted sum of the expected NPV over all future scenarios and the CVaR according to (4). It is assumed that after $t^{\star}$ periods, the upcoming future scenario $s^{\star}$ is known. Thus, the supply network configuration determined in the first stage is fixed for the periods $t=1,\ldots,t^{\star}$, i.e., the respective discrete variables are fixed for those periods, whereas the remaining discrete variables are not fixed. It is worth noting, that it is not mandatory to fix the real-valued decision variables, since they have no influence on subsequent periods. Hence, a future-scenario-specific variant of the RSNDPMC-PLA, called RSNDPMC-PLA${}_{s}$, is formulated. The RSNDPMC-PLA${}_{s}$ resembles the RSNDPMC-PLA except that it omits the CVaR-specific constraints (5) and (6). In place of the original objective function (4), a future scenario-specific $\text{NPV}_{s}$ according to (4a) to (4i) is maximized in the second stage. Furthermore, slack variables are incorporated into the $\beta$-service-level constraints (8) to always enable a mathematically feasible solution. However, these additional variables are penalized in the objective function. Thus, by solving the RSNDPMC-PLA${}_{s}$ for the upcoming future scenario $s^{\star}$, an adapted future scenario-specific supply network configuration is generated for the periods $t=t^{\star}+1,\ldots,T$ in the second stage. This two-stage stochastic programming approach is outlined in Algorithm 1. 
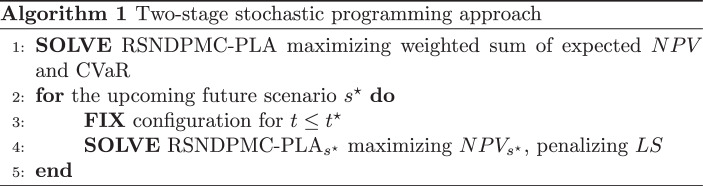


Note that we focus on the supply network configuration in the two-stage stochastic programming approach. In a subsequent tactical or operational planning step, production and transportation quantities can be further adapted to be in line with the determined capacities of the supply network configuration.

## Numerical results

### Description of the test design

Two problem classes (PCs) are defined for our numerical investigation. These PCs differ with respect to the number of components $C$, vendors $V$, products $P$, facilities $F$, module types $M$, retailers $R$ and periods $T$, as shown in Table 2.

**Table 2:** Sizes of problem classes

	$C$	$V$	$P$	$F$	$M$	$R$	$T$	#TI
**PC 1**	5	10	5	6	5	6	6	40
**PC 2**	5	10	10	10	10	6	12	40

There are two spatially different activity regions; each contains half the vendors, facilities and retailers. On average, demand is forecast to shift from one activity region to the other along the planning horizon. For our numerical study, three future scenarios (low, normal and high demand) are considered, as described in 2. For the definition of test instances (TIs), two parameters—each with two different values—are varied. The coefficient of variation, $\textit{VC}^{d}$, which is related to uncertain demand, corresponds to $\textit{VC}^{d}\in\{0.3,\,0.5\}$, and two target $\beta$-service levels, where $\beta\in\{0.9,\,0.95\}$, are used. The variation in both parameter values results in 4 parameter settings. For each setting, 10 TIs are randomly generated. Thus, each PC consists of 40 different TIs. The TIs are described in detail in 2.

Sahling and Kayser (2016) noted that 10 linear segments already ensure a sufficient accuracy of the approximation approach without a substantial increase in the numerical effort. Thus, $L=10$ segments are also used in our numerical experiments. For the two-stage stochastic programming approach, the supply network configuration is fixed after $t^{\star}=3$ for PC 1 and $t^{\star}=5$ for PC 2 in the second stage.

For the numerical analysis, an additional variant of the RSNDPMC-PLA is defined, where relocations of modular capacities are omitted. In this so-called RSNDPMC-NRL, the corresponding integer variables $N^{ \textit{Mreloc}}_{ \textit{mff't}}$ are fixed to zero. Due to this fixation, the consideration of relocatable modules almost doubles the number of discrete variables in the case of PC 1 and more than triples the number of discrete variables in the case of PC 2.

Numerical experiments were conducted on the cluster TANE of the LUIS in Hannover using 2 parallel threads, each with a 2.93 GHz processor and a maximum of 16 GB of RAM. The described variants of the RSNDPMC-PLA are implemented in GAMS 24.5.4, and each TI is solved to (sub)optimality using CPLEX 12.6. The optimization process is terminated by CPLEX if a given time limit *TLim* is reached.

In Table 3, the solution qualities of the RSNDPMC-PLA, the RSNDPMC-PLA${}_{s}$, and the RSNDPMC-NRL are reported for PC 1 and PC 2.

**Table 3:** Solution qualities of the RSNDPMC-PLA, -PLA${}_{s}$, and -NRL

		TLim	TCPU	OptSol
		[s]	[s]	[%]
RSNDPMC-PLA	**PC 1**	21,600	6,311	80.0
	**PC 2**	43,200	41,348	5.0
RSNDPMC-PLA${}_{s}$	**PC 1**	3,600	68	98.8
	**PC 2**	3,600	2,721	37.5
RSNDPMC-NRL	**PC 1**	21,600	751	100.0
	**PC 2**	43,200	38,580	17.5

*TLim* Given time limit, *TCPU* Average solution time, *OptSol* Percentage of optimally solved TIs

Notably, with an average gap of less than 0.05% in the case of PC 1 and less than 0.24% for PC 2, (near) optimal solutions are obtained by CPLEX for all considered TIs within the given time limit.

### Analysis of the relocatability of modules

**Fig. 4:**
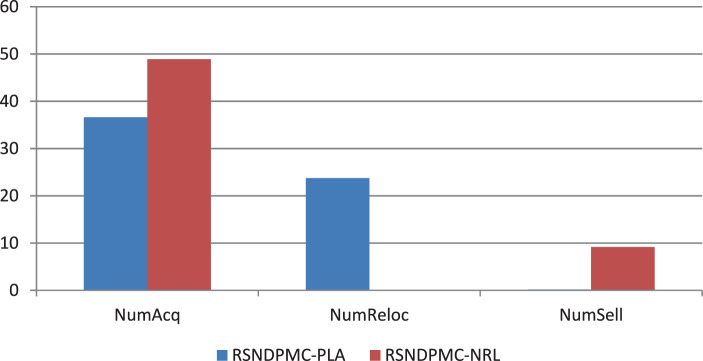
Number of acquired, relocated and sold modules for PC 1 after the first stage

**Fig. 5:**
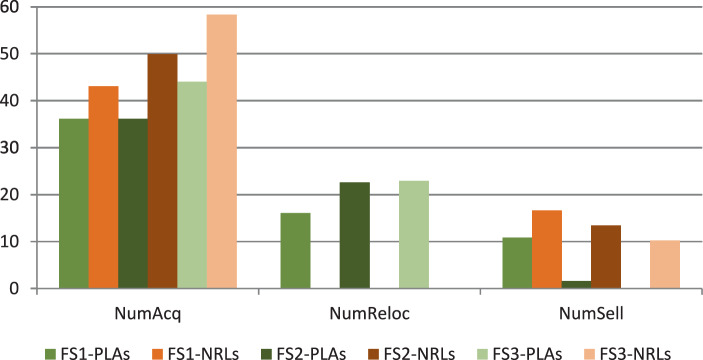
Number of acquired, relocated and sold modules for PC 1 for the two-stage approach

For the numerical study, we use all TIs described in Sect. 5.1. To demonstrate the impact of the possibility of relocating modules among production facilities, we analyze the average number of newly acquired modules *NumAcq*, relocations *NumReloc*, and sold modules *NumSell* for four variants of the RSNDPMC, i.e., RSNDPMC-PLA, RSNDPMC-PLA${}_{s}$, RSNDPMC-NRL and RSNDPMC-NRL${}_{s}$. The last model variant follows the two-stage approach described in Sect. 4.2, but relocations are not permitted. The results for PC 1 are presented in Figs. 4 and 5. Furthermore, the results for PC 2 are quite similar, and only the overall dimensions differ.

These results show that for RSNDPMC-PLA, where relocations are possible, the average number of acquired modules can be reduced by more than 25% for PC 1 and by more than 17% for PC 2 compared to RSNDPMC-NRL, which does not include relocations. This is an important result when considering ecological effects. The use of relocatable modular capacities leads to a substantial decrease in the number of required modules and therefore to protection of the resources required to build new modules.

The introduction of the two-stage approach leads to different effects according to the respective future scenario. In the case that after $t^{\star}$ periods, it becomes evident that future scenario FS1 has arisen, i.e., an overall low demand, and the (using the RSNDPMC-PLA) originally planned number of modules to be acquired was overestimated. Therefore, in this future scenario, modules are sold. In the case of future scenario FS2 (i.e., an overall normal demand), almost no changes are made to the structure derived by the RSNDPMC-PLA. This shows that for the FS2, the future scenario with the highest probability, the RSNDPMC-PLA already gives good results. For future scenario FS3 (i.e., an overall high demand), we observe a different effect. In this case, the number of modules to be acquired was underestimated. The number of acquired modules thus increases to meet the increased demand, and modules are not sold. Note that in the version where no relocations are possible, the number of acquired modules, as well as the number of sold modules, exceeds the respective numbers when relocations are allowed for all future scenarios.

To analyze the impact of the difference in the number of acquired and relocated modules on the NPV, a simulation study is conducted with 1,000 replications. For each replication, different realizations of the uncertain demand are randomly generated for each future scenario. To evaluate this simulation, all the discrete variables are fixed according to their values after the optimization, i.e., all strategic decisions regarding

- the location of production facilities, i.e., establishing, running and closing,
- and the assignment of modular capacities, i.e., the acquisition, holding, and selling of modules and, if applicable, their relocation

cannot be modified. However, all real-valued decision variables regarding transportation quantities are not fixed. We also use slack variables in the $\beta$-service-level constraints (8) to ensure a mathematically feasible solution. These additional variables are penalized in the objective function. The remaining linear program is solved to optimality using CPLEX for each replication. Note that in the case of the RSNDPMC-PLA${}_{s}$, the future scenario-specific supply network configuration of the second stage is used.

The results of this simulation show that the NPV increases on average by more than 5% for PC 1 and by more than 3% for PC 2 in the case of RSNDPMC-PLA compared to RSNDPMC-NRL. After recourse, the RSNDPMC-PLA${}_{s}$ still generates an on average more than 5% higher NPV than the RSNDPMC-NRL${}_{s}$ for PC 1 and more than 2% higher NPV for PC 2.

### Simulation-based analysis of the robustness

As discussed above, the transportation and production quantities can be adapted in subsequent planning steps with respect to the supply network configuration derived by the described variants of the RSNDPMC, i.e., RSNDPMC-PLA, RSNDPMC-PLA${}_{s}$ and RSNDPMC-NRL. To analyze the robustness of the determined supply network configuration, the same simulation study is used as was presented in Sect. 5.2.

In Table 4, the portion *SimFeas* of those scenarios that are feasibly solved in the simulation, i.e., the incorporated slack variables are equal to zero for all product-retailer-period combinations $P\times R\times T$, is provided. Furthermore, we report the average portion $P\times R\times T-$*Vio* of product-retailer-period combinations for which the target $\beta$-service level is violated.

**Table 4:** Robustness of supply network design

		SimFeas	$P\times R\times T-$Vio
		[%]	[%]
RSNDPMC-PLA	**PC 1**	90.0	0.14
	**PC 2**	84.3	0.12
RSNDPMC-PLA${}_{s}$	**PC 1**	92.0	0.09
	**PC 2**	86.4	0.07
RSNDPMC-NRL	**PC 1**	91.8	0.10
	**PC 2**	84.2	0.12

**Fig. 6:**
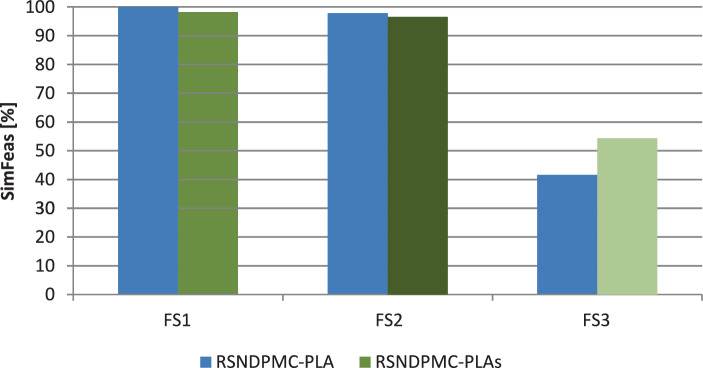
Percentage of feasibly solved simulation scenarios for the different future scenarios for PC 2

**Fig. 7:**
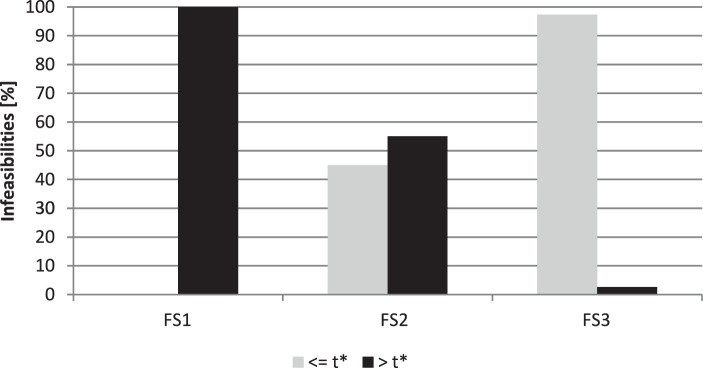
Percentage of infeasibilities arising before and after the recourse for PC 2

For all variants of the RSNDPMC, the derived supply network configuration meets the target $\beta$-service level in more than 82% of the simulation scenarios. However, in the cases where the target service level is not met, the $\beta$-service level is fulfilled for more than 99.8% of the product-retailer-period combinations. On average, when the supply network configuration is adjusted, an improvement can be achieved in terms of feasibility. It is, however, worth looking at the respective future scenarios separately in greater detail because the effects regarding feasibility vary. The results regarding the number of feasibly solved simulation scenarios are given exemplary for PC 2 in Fig. 6. In Fig. 7, the percentage of infeasibilities arising before and after the recourse are shown exemplarily for PC 2 again. The results for PC 1 differ only slightly.

As analyzed in Sect. 5.2, for future scenario FS1, i.e., when overall low demand occurs, the number of acquired modules was too high for the first $t^{\star}$ periods. Accordingly, the number of held modules is reduced after $t^{\star}$ periods. Therefore, for PC 1 and PC 2, a feasibility of nearly 100% can be achieved after the first-stage optimization for future scenario FS1. After the second stage (i.e., after adaptation), this feasibility decreases slightly. All infeasibilities arise after $t^{\star}$. This result shows that because fewer modules are in the system after adaptation, a very small number of infeasibilities occur that were covered by the extra modules in stage one and, therefore, did not arise. For future scenario FS2 (i.e., an overall normal demand), the supply network configuration hardly changes after the recourse; therefore, the feasibility is hardly influenced as well. A feasibility of more than 99% is reached for FS2 before and after the recourse for PC 1 and a feasibility of more than 96% for PC 2. Infeasibility occurs both before and after the recourse. For future scenario FS3 (i.e., an overall high demand), we found in Sect. 5.2 that the number of modules for the system generated in the first stage was too low. Therefore, in the second stage, additional modules are acquired after $t^{\star}$ to cover the high demand. For this future scenario, the first-stage configuration generates the smallest number of feasible solutions. Only 61.1% of the scenarios of FS3 can feasibly be solved for PC 1 and 41.6% for PC 2. The recourse can improve these results. After the recourse, 69.2% of the scenarios can feasibly be solved for PC 1 and 54.4% for PC 2. Because more than 97% of the infeasibilities arise in the periods before the recourse, this value cannot be improved further. This number shows that hardly any infeasibilities arise after the adaptation of the supply network configuration, and therefore, a very robust solution was found. Even in this case, only 0.31% and 0.24% of the product-retailer-period combinations are violated for PC 1 and PC 2, respectively.

The relocatability of modules does not considerably influence the number of feasible solutions. The infeasibilities depend on the provided module capacities at the facilities. The consideration of greater uncertainty, i.e., a higher value for $\textit{VC}^{d}$, leads to more robust solutions, i.e., the number of feasibly solved simulation scenarios increases. Greater uncertainty results in a supply network configuration with higher capacity allocations, i.e., more installed modules. However, greater maximum and average violations of the target service level are observable. The results of the $\beta$-service level show, that a higher service level yields a greater number of feasibly solvable simulation scenarios. In this case, more modules are also provided with higher capacity, where the maximum and average violations of the target service level are lower.

In summary, the simulation-based analysis shows that all variants of the RSNDPMC that consider uncertainty generate robust solutions, as shown by the high number of feasible solutions in the simulation study. By using the two-stage approach, this robustness can be increased further.

### The impact of $\alpha$ and $\psi$

**Fig. 8:**
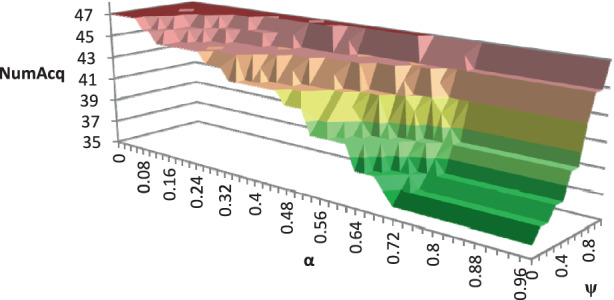
Average number of acquired modules for RSNDPMC-NRL regarding $\alpha$ and $\psi$

**Fig. 9:**
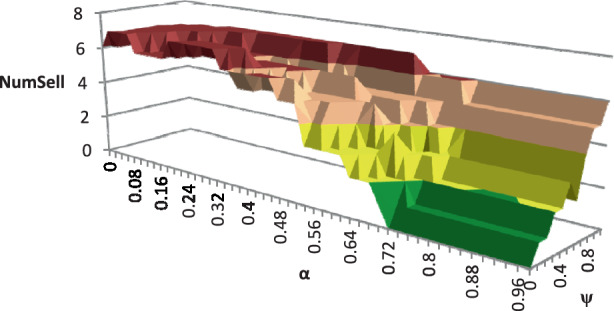
Average number of sold modules for RSNDPMC-NRL regarding $\alpha$ and $\psi$

**Fig. 10:**
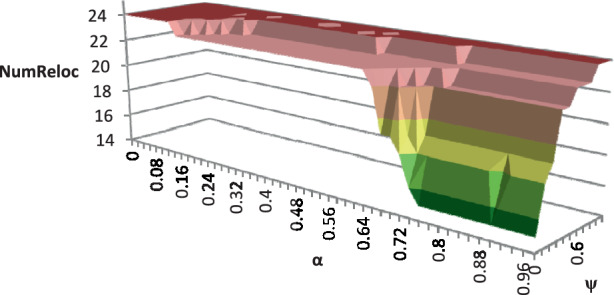
Number of relocated modules for RSNDPMC-PLA regarding $\alpha$ and $\psi$

To investigate the impact of the parameters $\alpha$, i.e., the probability related to the CVaR, and $\psi$, the weighting coefficient in the objective function (4), on the number of acquired modules and their relocation, we considered different values for $\alpha$ and $\psi$ for a TI from PC 1 that could be solved to optimality within the given time limit. We discovered that there is a significant impact on the number of acquired and sold modules for the RSNDPMC-NRL (see Figs. 8 and 9) and on the number of relocated modules for the RSNDPMC-PLA (see Fig. 10).

Fig. 8 shows that the number of acquired modules increases with decreasing values of $\alpha$. It is mentionable that the lowest NPV is generated in future scenario FS1 due to the lowest realized demand. Therefore, for an $\alpha$ close to 1, only the NPV of this worst scenario is considered in the CVaR-part of the objective function. Our study in Sect. 5.2 demonstrated that the number of necessary modules is smallest for this future scenario. Hence, to generate a high CVaR for an $\alpha$ close to 1, it is a good strategy to only acquire a small number of modules. As shown in Fig. 8, the number of acquired modules is smallest for an $\alpha$ close to 1. This means that, on the other hand, the more, i.e., $(1-\alpha)\cdot 100\%$, “bad” scenarios that are considered to calculate the CVaR, the more modules that are acquired to yield a better CVaR for the objective function. In this case, modules are acquired for being able to cover the high demand in future scenario FS3, thereby yielding a high NPV. The inverted effect is evident with the parameter $\psi$. With a decreasing value of $\psi$, the number of acquired modules also decreases. The smaller the value for $\psi$, the more weight is given to the CVaR in the objective function. This means that the decision maker is risk averse. Therefore, the investment for expensive modules is not made. In accordance with the number of acquired modules, the number of sold modules shows the same characteristics (see Fig. 9).

Fig. 10 shows that the effect on the number of relocated modules for the RSNDPMC-PLA is similar to the effects just described. With increasing values of $\alpha$, the number of relocated modules decreases, and the number decreases with decreasing values of $\psi$, i.e., with increasing risk aversion. However, the number of acquired modules for the RSNDPMC-PLA is hardly affected by $\alpha$ and $\psi$. This shows that in the case where relocations are possible, the expenses for the relocation of the modules strongly impact the objective value and therefore the reconfiguration of the supply network under uncertainty. Again, in the case whereby $\alpha$ is close to 1, the CVaR resembles the NPV of future scenario FS1. Due to the low demand in this scenario, it is not necessary to relocate many modules to fulfill the low demand. Relocating more modules than necessary only yields high payments for the relocation of the modules, while there is no demand and therefore no incoming payments for products produced on these modules. Hence, for an $\alpha$ close to 1, the number of relocated modules approaches the small number of relocations in FS1. On the other hand, if $\alpha$ is close to 0, the number of relocated modules resembles the relocation structure of FS3. Therefore, many modules are relocated—following the shift in demand—to avoid high payments for transporting the high number of products over a great distance to the retailer. The same effects observed with the RSNDPMC-PLA are noticeable for the RSNDPMC-PLA${}_{s}$.

## Conclusion

In this paper, a new model formulation for robust supply network reconfiguration is presented. In this model formulation, the concept of relocatable modular capacities is incorporated for the first time in the presence of twofold uncertain demand information. Due to the explicit consideration of uncertain demand and the resulting induced risk, a generic nonlinear model formulation arises. Nonlinear functions of expected lost sales are approximated by piecewise linear functions according to Helber et al. (2013). The solution of the resulting mixed-integer linear program results in a robust supply network configuration. Furthermore, a two-stage stochastic programming approach is proposed that enables a future scenario-specific modification of the determined supply network configuration.

In our numerical studies, we have observed that the consideration of relocatable modular capacities has a significant effect on the supply network structure and on the resulting NPV. The possibility of relocating modules provides the opportunity to significantly decrease the number of newly acquired modules, which results in a measurable increase in the NPV and has an as yet unquantified positive ecological effect. Furthermore, the number of newly acquired modules and the number of relocations increase under higher target service levels and higher uncertainty, i.e., with a higher coefficient of variation. The recourse enables an even better adaptation to the demand situation, resulting in even better values of NPV.

Additionally, our studies have revealed that the robustness of the supply network design increases with higher target service levels and higher coefficients of variation. Again, the consideration of the two-stage approach with recourse provides better results, this time regarding feasibility.

The analysis of the impact of the parameter $\alpha$, i.e., the probability related to the CVaR, and $\psi$, the weighting coefficient in the objective function, on the network configuration has shown that both parameters have a great impact. With an $\alpha$ close to 1, the configuration approaches the optimal configuration of future scenario FS1, while a small $\alpha$ close to 0 leads to a configuration approaching the optimal configuration for FS3. For a decreasing value of $\psi$, the decision maker is increasingly risk averse, and large investments for the acquisition and relocation of modules are not made.

Future research should address a scenario-specific retail price. Future scenarios may affect the retail price as well; thus, it becomes uncertain. A demand function must be assumed to determine the scenario-specific retail price. Hence, further nonlinearities occur in the model formulation. An uncertain retail price may also have an effect on both the expected net present value and the supply network configuration. Furthermore, a life-cycle assessment should be processed to evaluate the environmental impact of the relocatable modules.

## Appendix

### Notation of the RSNDPMC

**Table:**

Indices and Sets:	
$c\in\mathcal{C}$	Set of components $(c=1,\ldots,C)$
$f,f^{\prime}\in\mathcal{F}$	Set of potential production facilities $(f=1,\ldots,F)$
$l\in\mathcal{L}$	Set of linearization segments $(l=0,\ldots,L)$
$m\in\mathcal{M}$	Set of module types $(m=1,\ldots,M)$
$p\in\mathcal{P}$	Set of end products $(p=1,\ldots,P)$
$r\in\mathcal{R}$	Set of retailers $(r=1,\ldots,R)$
$s\in\mathcal{S}$	Set of future scenarios $(s=1,\ldots,S)$
$t,\,\tau\in\mathcal{T}$	Set of periods $(t=1,\ldots,T)$
$v\in\mathcal{V}$	Set of vendors $(v=1,\ldots,V)$
Subsets:	
$\mathcal{M}_{p}\subseteq\mathcal{M}$	Set of module types that can produce end product $p$
$\mathcal{P}_{c}\subseteq\mathcal{P}$	Set of end products requiring component $c$
$\mathcal{P}_{m}\subseteq\mathcal{P}$	Set of end products producible on module type $m$
$\mathcal{V}_{c}\subseteq\mathcal{V}$	Set of vendors providing component $c$
Parameters:	
$\alpha$	Probability related to CVaR
$\beta_{ \textit{pr}}$	Target $\beta$-service level for end product $p$ at retailer $r$
$\Delta^{ \textit{LS}}_{ \textit{prtls}}$	Slope for expected lost sales of end product $p$ at retailer $r$ in period $t$ for segment $l$ and future scenario $s$
$\pi_{s}$	Probability of future scenario $s$
$\psi$	Level of risk aversion
$\textit{cap}^{ \textit{Cmax}}_{ \textit{cv}}$	Maximum ordering capacity of component $c$ at vendor $v$ per period
$\textit{cap}^{ \textit{Cmin}}_{ \textit{cv}}$	Minimum ordering capacity of component $c$ at vendor $v$ per period
$\textit{cap}^{ \textit{Mmax}}_{ \textit{m}}$	Total maximum production capacity per module of type $m$ per period
$\textit{cf}^{ \textit{Macqu}}_{ \textit{mft}}$	Capacity consumption factor of installing a module of type $m$ after acquisition at facility $f$ in period $t$
$\textit{cf}^{ \textit{Mreloc}}_{ \textit{mff't}}$	Capacity consumption factor of relocating one module of type $m$ from facility $f$ and installing it at facility $f^{\prime}$ in period $t$ including transportation lag
$\textit{cf}^{ \textit{Pprod}}_{ \textit{pmft}}$	Capacity consumption factor of manufacturing one unit of end product $p$ on a module of type $m$ at facility $f$ in period $t$
$\textit{cq}^{ \textit{Pprod}}_{ \textit{prtls}}$	Maximum production quantity of end product $p$ for retailer $r$ in period $t$ for segment $l$ cumulated over all facilities for future scenario $s$
$\textit{els}_{ \textit{prtls}}$	Expected lost sales of end product $p$ at retailer $r$ in period $t$ for segment $l$ and future scenario $s$
$i^{ \textit{wacc}}_{t}$	Company-specific rate of interest in period $t$
$\textit{pay}^{ \textit{close}}_{ \textit{ft}}$	Payment for closing facility $f$ in period $t$ (outgoing payments for the closing process – incoming payments from the selling process)
$\textit{pay}^{ \textit{Cacqu}}_{ \textit{cvt}}$	Payment for acquiring one unit of component $c$ from vendor $v$ in period $t$
$\textit{pay}^{ \textit{Corder}}_{ \textit{cvt}}$	Payment for ordering component $c$ from vendor $v$ in period $t$
$\textit{pay}^{ \textit{Ctrans}}_{ \textit{cvft}}$	Payment for transporting one unit of component $c$ from vendor $v$ to facility $f$ in period $t$
$\textit{pay}^{ \textit{est}}_{ \textit{ft}}$	Payment for establishing facility $f$ in period $t$
$\textit{pay}^{ \textit{Macqu}}_{ \textit{mft}}$	Payment for acquiring and installing one module of type $m$ in facility $f$ in period $t$
$\textit{pay}^{ \textit{Mhold}}_{ \textit{mft}}$	Payment for holding one module of type $m$ in facility $f$ in period $t$
$\textit{pay}^{ \textit{Mreloc}}_{ \textit{mff't}}$	Payment for relocating a module of type $m$ from facility $f$ and installing it at facility $f^{\prime}$ in period $t$
$\textit{pay}^{ \textit{Msell}}_{ \textit{mf}}$	(Incoming) payment for selling a module of type $m$ from facility $f$ (including outgoing payments for deinstalling the module)
$\textit{pay}^{ \textit{open}}_{ \textit{ft}}$	Payment for open facility $f$ in period $t$
$\textit{pay}^{ \textit{Pprod}}_{ \textit{pmft}}$	Payment for manufacturing one unit of end product $p$ on a module of type $m$ at facility $f$ in period $t$
$\textit{pay}^{ \textit{Psell}}_{ \textit{prt}}$	(Incoming) payment for selling one unit of product $p$ at retailer $r$ in period $t$
$\textit{pay}^{ \textit{Ptrans}}_{ \textit{pfrt}}$	Payment for transporting one unit of end product $p$ from facility $f$ to retailer $r$ in period $t$
$\textit{sp}_{m}$	Required space by one module of type $m$
$\textit{SP}^{ \textit{max}}_{ \textit{ft}}$	Maximum available space at facility $f$ in period $t$
$u_{ \textit{cp}}$	Number of units of component $c$ required to produce one unit of end product $p$
Random variables:	
$\mathbf{D}_{ \textit{prts}}$	Demand of end product $p$ at retailer $r$ in period $t$ for future scenario $s$
$\mathbf{LS}_{ \textit{prts}}$	Lost sales of end product $p$ at retailer $r$ in period $t$ for future scenario $s$
Binary decision variables:	
$\varphi^{ \textit{close}}_{ \textit{ft}}$	1 if facility $f$ is closed at the beginning of period $t$; 0 otherwise
$\varphi^{ \textit{est}}_{ \textit{ft}}$	1 if facility $f$ is established at the beginning of period $t$; 0 otherwise
$\varphi^{ \textit{open}}_{ \textit{ft}}$	1 if facility $f$ is open in period $t$; 0 otherwise
$\vartheta^{ \textit{Corder}}_{ \textit{cvts}}$	1 if component $c$ is ordered at vendor $v$ in period $t$ for future scenario $s$; 0 otherwise
Positive integer decision variables:	
$N^{ \textit{Macqu}}_{ \textit{mft}}$	Number of modules of type $m$ acquired at facility $f$ at the beginning of period $t$
$N^{ \textit{Mhold}}_{ \textit{mft}}$	Number of modules of type $m$ held at facility $f$ in period $t$
$N^{ \textit{Mreloc}}_{ \textit{mff't}}$	Number of modules of type $m$ relocated from facility $f$ to facility $f^{\prime}$ at the beginning of period $t$
$N^{ \textit{Msell}}_{ \textit{mft}}$	Number of modules of type $m$ sold at facility $f$ at the beginning of period $t$
Positive real-valued decision variables	
$\omega_{s}$	Auxiliary variable for the calculation of the CVaR for future scenario $s$
$q^{ \textit{Ctrans}}_{ \textit{cvfts}}$	Transportation quantity of component $c$ from vendor $v$ to facility $f$ in period $t$ for future scenario $s$
$q^{ \textit{Pprod}}_{ \textit{pmfrts}}$	Production quantity of end product $p$ on any module of type $m$ at facility $f$ for retailer $r$ in period $t$ for future scenario $s$
$w^{ \textit{Pprod}}_{ \textit{prtls}}$	Production quantity of end product $p$ for retailer $r$ in period $t$ in segment $l$ cumulated over all facilities for future scenario $s$
Real-valued decision variables	
$\omega_{0}$	Auxiliary variable for the calculation of the CVaR
CVaR	Conditional value-at-risk
$\widetilde{{\mathbb{E}}}[\mathbf{LS}_{ \textit{prts}}]$	Approximated expected lost sales of end product $p$ at retailer $r$ in period $t$ for future scenario $s$
$\text{NPV}_{s}$	Net present value of future scenario $s$

### Description of the test instances

The described procedure for the generation of our test instances depends primarily on Sahling and Kayser (2016). To simulate a shift in demand, as further described below, two spatially different activity regions are defined. The coordinates of each vendor $v$, production facility $f$ and retailer $r$ are defined randomly in two quadratic grids. Half of the vendors, facilities and retailers are generated in the grid $[0,100]\times[0,100]$ using a uniform distribution; see Melkote and Daskin (2001). The other half is generated in the grid $[300,400]\times[0,100]$ using a uniform distribution. We define the region in the grid $[0,100]\times[0,100]$ to be a region with decreasing demand over time, whereas the region in the grid $[300,400]\times[0,100]$ is assumed to have an increasing demand over time. The distance between vendors and facilities $\textit{dist}_{ \textit{vf}}$, between two different facilities $\textit{dist}_{ \textit{ff'}}$ and between facilities and retailers $\textit{dist}_{ \textit{fr}}$ are based on Euclidean distance measures. The internal rate of interest $i^{ \textit{wacc}}_{t}$ is equal to 8%. To consider risk propensity, we assume a risk-neutral decision maker as $\alpha=0.5$ and $\psi=0.5$.

In the following, a continuous uniform distribution between $a$ and $b$ is described by the expression $U[a,b]$, whereas a discrete uniform distribution between $a$ and $b$ is denoted by $U\{a,b\}$.

The components are randomly assigned to vendors such that each component $c$ is provided by 3 to 6 vendors, i.e., ${\lvert}\mathcal{V}_{c}{\rvert}\sim U\{3,6\}$. Each vendor $v$ provides 1 to 3 components. Furthermore, the components are also randomly assigned to end products such that end product $p$ contains 1 to 3 components, and each component $c$ is required by 3 to 5 products, i.e., ${\lvert}\mathcal{P}_{c}{\rvert}\sim U\{3,5\}$. For each $p\in\mathcal{P}_{c}$, the respective $u_{ \textit{cp}}$ is set to 1. The set of required components $\mathcal{C}_{p}$ for end product $p$ contains those components $c$ with $u_{ \textit{cp}}> 0$. To generate several module types with different product portfolios, end products are randomly assigned to module types such that each end product $p$ can be manufactured by at least two module types $m$. Furthermore, each module type $m$ can manufacture at least two end products $p$.

The scenario-specific time series of the expected demand ${\mathbb{E}}[\mathbf{D}_{ \textit{prts}}]$ is generated as follows. We first estimate the maximum expected demand ${\mathbb{E}}[\mathbf{D}^{ \textit{max}}_{ \textit{p}}]$ for each end product $p$, where ${\mathbb{E}}[\mathbf{D}^{ \textit{max}}_{ \textit{p}}]\sim U\{500,700\}$. To consider retailer dependencies, the maximum expected demand ${\mathbb{E}}[\mathbf{D}^{ \textit{max}}_{ \textit{pr}}]$ of end product $p$ at retailer $r$ is calculated by ${\mathbb{E}}[\mathbf{D}^{ \textit{max}}_{ \textit{pr}}]=U[0.75,1.25]\cdot{\mathbb{E}}[\mathbf{D}^{ \textit{max}}_{ \textit{p}}]$. To simulate the shift in the period-specific expected demand ${\mathbb{E}}[\mathbf{D}_{ \textit{prt}}]$, a linear function is used. In the region of decreasing demand over the planning horizon, the ${\mathbb{E}}[\mathbf{D}_{ \textit{prt}}]$ decreases from ${\mathbb{E}}[\mathbf{D}^{ \textit{max}}_{ \textit{pr}}]$ linearly over time until it nearly vanishes in the last planning period. Otherwise, in the region of increasing demand, the expected demand ${\mathbb{E}}[\mathbf{D}_{ \textit{prt}}]$ increases linearly from nearly no demand until ${\mathbb{E}}[\mathbf{D}^{ \textit{max}}_{ \textit{pr}}]$ is reached in the last planning period. In the next step, the three future scenarios $s\in\{ \textit{low, normal, high}\}$ with their respective demand factors $\xi_{ \textit{low}}=0.7$, $\xi_{ \textit{normal}}=1.0$ and $\xi_{ \textit{high}}=1.3$ are incorporated. The probability of the normal scenario is $\pi_{ \textit{normal}}=0.5$, and the probability of the remaining scenarios is $\pi_{ \textit{low/high}}=0.25$. To account for slight fluctuations in the linear demand curve, the scenario-specific expected demand ${\mathbb{E}}[\mathbf{D}_{ \textit{prts}}]$ can then be derived from a normal distribution with mean $\xi_{s}\cdot{\mathbb{E}}[\mathbf{D}_{ \textit{prt}}]$ and standard deviation $U[0.1,0.2]\cdot\xi_{s}\cdot{\mathbb{E}}[\mathbf{D}_{ \textit{prt}}]$ for each end product $p$, retailer $r$ and period $t$.

Using this scenario-specific expected demand ${\mathbb{E}}[\mathbf{D}_{ \textit{prts}}]$ and the given coefficient of variation $\textit{VC}^{d}$, the uncertain demand $\mathbf{D}_{ \textit{prts}}$ is normally distributed with mean ${\mathbb{E}}[\mathbf{D}_{ \textit{prts}}]$ and standard deviation $\sigma_{ \textit{prt}}= \textit{VC}^{d}\cdot{\mathbb{E}}[\mathbf{D}_{ \textit{prts}}]$.

To allow different dimensions of vendors, we define a capacity factor $\textit{cap}^{ \textit{Cfac}}_{ \textit{cv}}$ for each vendor $v$ and component $c$, where

$$\textit{cap}^{ \textit{Cfac}}_{ \textit{cv}}=\frac{U\{1,{\lvert}\mathcal{V}_{c}{\rvert}\}}{{\lvert}\mathcal{V}_{c}{\rvert}}.$$

The capacity $\textit{cap}^{ \textit{Cmax}}_{ \textit{cv}}$ can be determined using the average expected demand $\overline{ \textit{AD}}_{ \textit{p}}$ of end product $p$ as follows:

$$\textit{cap}^{ \textit{Cmax}}_{ \textit{cv}}= \textit{cap}^{ \textit{Cfac}}_{ \textit{cv}}\cdot\sum_{p\in\mathcal{P}_{c}}u_{ \textit{cp}}\cdot\overline{ \textit{AD}}_{ \textit{p}}\cdot(1+ \textit{VC}^{d})$$

with

$$\overline{ \textit{AD}}_{ \textit{p}}=\sum_{r\in\mathcal{R}}\sum_{t}\frac{{\mathbb{E}}[\mathbf{D}_{ \textit{prt}}]}{T}.$$

In this way, it is ensured that components are available in a sufficient number and do not describe a bottleneck. The minimum capacities $\textit{cap}^{ \textit{Cmin}}_{ \textit{cv}}$ are assumed to be zero.

For each module type $m$, we generate a capacity factor $\textit{cap}^{ \textit{Mfac}}_{m}$ to enable different sizes for module types, where

$$\textit{cap}^{ \textit{Mfac}}_{m}=\frac{{\lvert}\mathcal{P}_{m}{\rvert}}{\sum_{ \textit{m'}}{\lvert}\mathcal{P}_{ \textit{m'}}{\rvert}}.$$

The capacity consumption factor of manufacturing one unit of end product $p$ on module type $m$ at facility $f$ in period $t$ is determined by $\textit{cf}^{ \textit{Pprod}}_{ \textit{pmft}}=U\{1,2\}$. Using this consumption factor, the capacity $\textit{cap}^{ \textit{Mmax}}_{m}$ can be derived as follows:

$$\textit{cap}^{ \textit{Mmax}}_{ \textit{m}}=0.1\cdot \textit{cap}^{ \textit{Mfac}}_{m}\cdot\sum_{p\in P_{m}}{\overline{ \textit{AD}}_{ \textit{p}}\cdot(1+ \textit{VC}^{d})\cdot\frac{\sum_{m^{\prime}\in M_{p}}\sum_{f}\sum_{t}cf^{ \textit{Pprod}}_{ \textit{pm'ft}}}{{\lvert}\mathcal{M}_{p}{\rvert}\cdot{\lvert}\mathcal{F}{\rvert}\cdot{\lvert}\mathcal{T}{\rvert}}},$$

to force the acquisition of more than one module. The capacity consumption due to the acquisition of module $m$ at facility $f$ in period $t\,cf^{ \textit{Macqu}}_{ \textit{mft}}=0.1\cdot \textit{cap}^{ \textit{Mmax}}_{ \textit{m}}$. Depending on the distance between two facilities in relation to the distances among all facilities, the consumption factor due to the relocation of a module can be calculated as follows:

$$cf^{ \textit{Mreloc}}_{ \textit{mff't}}=cf^{ \textit{Macqu}}_{ \textit{mft}}\cdot(1+\frac{ \textit{dist}_{ \textit{ff'}}}{\sum_{ \textit{f''}}\sum_{ \textit{f'''> f''}} \textit{dist}_{ \textit{f''f'''}}}).$$

The required space per module type $\textit{sp}_{m}=100\cdot \textit{cap}^{ \textit{Mfac}}_{m}$.

For each facility $f$, we randomly generate a capacity factor $\textit{cap}^{ \textit{Ffac}}_{f}$, where

$$\textit{cap}^{ \textit{Ffac}}_{f}=\frac{U\{1,F\}}{F},$$

leading to differently dimensioned facilities. Furthermore, more than one production facility has to be established. Based on the required space per module, we generate the maximum available space at a facility as follows:

$$\textit{SP}^{ \textit{max}}_{ \textit{ft}}=(1+ \textit{cap}^{ \textit{Ffac}}_{f})\cdot\frac{\sum_{m} \textit{sp}_{m}\cdot\sum_{p}\overline{ \textit{AD}}_{ \textit{p}}\cdot(1+ \textit{VC}^{d})}{\sum_{m} \textit{cap}^{ \textit{Mfac}}_{m}}.$$

In Table 5, we describe the determination of several payment parameters similar to Cordeau et al. (2006), Cortinhal and Captivo (2003), and Thanh et al. (2008).

**Table 5:** Determination of the parameters for payments

Parameter		Value
$\textit{pay}^{ \textit{close}}_{ \textit{ft}}$	=	$2\cdot U[0.9,1.1]\cdot \textit{pay}^{ \textit{open}}_{ \textit{ft}}\cdot\sqrt{(1+ \textit{cap}^{ \textit{Ffac}}_{f})\cdot\sum_{p}\overline{ \textit{AD}}_{ \textit{p}}\cdot(1+ \textit{VC}^{d})}$
$\textit{pay}^{ \textit{Cacqu}}_{ \textit{cvt}}$	=	$U[5,10]\cdot U[\max\{0.5;1- \textit{cap}^{ \textit{Cfac}}_{ \textit{cv}}\},1]$
$\textit{pay}^{ \textit{Corder}}_{ \textit{cvt}}$	=	$U[1,10]\cdot\sqrt{ \textit{cap}^{ \textit{Cmax}}_{ \textit{cv}}}$
$\textit{pay}^{ \textit{Ctrans}}_{ \textit{cvft}}$	=	$0.05\cdot \textit{dist}_{ \textit{vf}}$
$\textit{pay}^{ \textit{est}}_{ \textit{ft}}$	=	$3\cdot U[0.9,1.1]\cdot \textit{pay}^{ \textit{open}}_{ \textit{ft}}\cdot\sqrt{(1+ \textit{cap}^{ \textit{Ffac}}_{f})\cdot\sum_{p}\overline{ \textit{AD}}_{ \textit{p}}\cdot(1+ \textit{VC}^{d})}$
$\textit{pay}^{ \textit{Macqu}}_{ \textit{mft}}$	=	$U[100,000,500,000]\cdot(1+ \textit{cap}^{ \textit{Mfac}}_{m})$
$\textit{pay}^{ \textit{Mhold}}_{ \textit{mft}}$	=	$0.2\cdot \textit{pay}^{ \textit{Macqu}}_{ \textit{mft}}$
$\textit{pay}^{ \textit{Mreloc}}_{ \textit{mff't}}$	=	$0.05\cdot \textit{pay}^{ \textit{Macqu}}_{ \textit{mft}}\cdot(1+\frac{ \textit{dist}_{ \textit{ff'}}}{\sum_{ \textit{f''}}\sum_{ \textit{f'''> f''}} \textit{dist}_{ \textit{f''f'''}}})$
$\textit{pay}^{ \textit{Msell}}_{ \textit{mf}}$	=	$0.1\cdot\frac{\sum_{t} \textit{pay}^{ \textit{Macqu}}_{ \textit{mft}}}{T}$
$\textit{pay}^{ \textit{open}}_{ \textit{ft}}$	=	$U[10,50]\cdot\sqrt{(1+ \textit{cap}^{ \textit{Ffac}}_{f})\cdot\sum_{p}\overline{ \textit{AD}}_{ \textit{p}}\cdot(1+ \textit{VC}^{d})}$
$\textit{pay}^{ \textit{Ptrans}}_{ \textit{pfrt}}$	=	$\sum_{c\in\mathcal{C}_{p}}u_{ \textit{cp}}\cdot \textit{dist}_{ \textit{fr}}$

Note that the following calculations for the different payments take the size of the facility and/or module into account. The production payments $\textit{pay}^{ \textit{Pprod}}_{ \textit{pmft}}$ of end product $p$ on module type $m$ at facility $f$ in period $t$ are proportional to the average payments $\overline{ \textit{pay}_{p}^{ \textit{C}}}$ for acquiring all required components for end product $p$, i.e.,

$$\overline{ \textit{pay}_{p}^{ \textit{C}}}=\sum_{ \textit{c}}u_{ \textit{cp}}\cdot\frac{\sum_{v\in\mathcal{V}_{c}}\sum_{t} \textit{pay}^{ \textit{Cacqu}}_{ \textit{cvt}}}{{\lvert}\mathcal{V}_{c}{\rvert}\cdot T}.$$

Based on $\overline{ \textit{pay}_{p}^{ \textit{C}}}$, the production payments $\textit{pay}^{ \textit{Pprod}}_{ \textit{pmft}}$ are defined as follows:

$$\textit{pay}^{ \textit{Pprod}}_{ \textit{pmft}}=\overline{ \textit{pay}_{p}^{ \textit{C}}}\cdot U\left[\max\left\{0.5,1-\frac{ \textit{cap}_{m}^{ \textit{Mfac}}}{\sum_{ \textit{m'}} \textit{cap}_{ \textit{m'}}^{ \textit{Mfac}}}\right\},1\right].$$

For a better understanding of the following calculations, we refrain from reducing fractions. To determine the (incoming) payments $\textit{pay}^{ \textit{Psell}}_{ \textit{prt}}$, it is essential to estimate the retailer-specific mean (outgoing) payments per unit of end product $p$ in each period $t$ in advance. Therefore, in the first step, the mean payments for ordering ($\overline{ \textit{pay}^{ \textit{Cacqu}}_{ \textit{pt}}}$) and transporting ($\overline{ \textit{pay}^{ \textit{Ctrans}}_{ \textit{pt}}}$) components and the mean payments for producing ($\overline{ \textit{pay}^{ \textit{Pprod}}_{ \textit{pt}}}$) and transporting ($\overline{ \textit{pay}^{ \textit{Ptrans}}_{ \textit{prt}}}$) end products are evaluated for one unit of end product $p$ as follows:

18 $$\overline{ \textit{pay}^{ \textit{Cacqu}}_{ \textit{pt}}}=\sum_{c}u_{ \textit{cp}}\cdot\sum_{v\in\mathcal{V}_{c}}\frac{ \textit{pay}^{ \textit{Cacqu}}_{ \textit{cvt}}}{{\lvert}\mathcal{V}_{c}{\rvert}},$$

19 $$\overline{ \textit{pay}^{ \textit{Ctrans}}_{ \textit{pt}}}=0.5\cdot\sum_{c}u_{ \textit{cp}}\cdot\left(\frac{\displaystyle\sum_{v\leq\left\lceil\frac{{\lvert}\mathcal{V}_{c}{\rvert}}{2}\right\rceil}\sum_{f\leq\left\lceil\frac{{\lvert}\mathcal{F}{\rvert}}{2}\right\rceil} \textit{pay}^{ \textit{Ctrans}}_{ \textit{cvft}}}{\frac{{\lvert}\mathcal{V}_{c}{\rvert}}{2}\cdot\frac{{\lvert}\mathcal{F}{\rvert}}{2}}+\frac{\displaystyle\sum_{v> \left\lceil\frac{{\lvert}\mathcal{V}_{c}{\rvert}}{2}\right\rceil}\sum_{f> \left\lceil\frac{{\lvert}\mathcal{F}{\rvert}}{2}\right\rceil} \textit{pay}^{ \textit{Ctrans}}_{ \textit{cvft}}}{\frac{{\lvert}\mathcal{V}_{c}{\rvert}}{2}\cdot\frac{{\lvert}\mathcal{F}{\rvert}}{2}}\right),$$

20 $$\overline{ \textit{pay}^{ \textit{Pprod}}_{ \textit{pt}}}=\frac{\sum_{f}\sum_{m\in\mathcal{M}_{p}} \textit{pay}^{ \textit{Pprod}}_{ \textit{pmft}}}{{\lvert}\mathcal{F}{\rvert}\cdot{\lvert}\mathcal{M}_{p}{\rvert}},$$

21 $$\overline{ \textit{pay}^{ \textit{Ptrans}}_{ \textit{prt}}}=\frac{\sum_{f\leq\left\lceil\frac{{\lvert}\mathcal{F}{\rvert}}{2}\right\rceil} \textit{pay}^{ \textit{Ptrans}}_{ \textit{pfrt}}}{{\frac{{\lvert}\mathcal{F}{\rvert}}{2}}}\qquad\forall r\leq\left\lceil\frac{{\lvert}\mathcal{R}{\rvert}}{2}\right\rceil,$$

22 $$\overline{ \textit{pay}^{ \textit{Ptrans}}_{ \textit{prt}}}=\frac{\sum_{f> \left\lceil\frac{{\lvert}\mathcal{F}{\rvert}}{2}\right\rceil} \textit{pay}^{ \textit{Ptrans}}_{ \textit{pfrt}}}{{\frac{{\lvert}\mathcal{F}{\rvert}}{2}}}\qquad\forall r> \left\lceil\frac{{\lvert}\mathcal{R}{\rvert}}{2}\right\rceil.$$

Note that the overall mean payments for transporting components are calculated as the mean over both demand regions. The payments for transporting end products are calculated as the mean for each demand region. In the next step, we determine the mean payments $\overline{ \textit{pay}^{ \textit{Macqu}}_{ \textit{pt}}}$ for acquiring modules for each end product $p$:

23 $$\overline{ \textit{pay}^{ \textit{Macqu}}_{ \textit{pt}}}=\frac{\overline{ \textit{AD}}_{p}\cdot\frac{\sum_{m\in\mathcal{M}_{p}}\sum_{f}cf^{ \textit{Pprod}}_{ \textit{pmft}}}{{\lvert}\mathcal{M}_{p}{\rvert}\cdot{\lvert}\mathcal{F}{\rvert}}}{\frac{\sum_{m\in\mathcal{M}_{p}} \textit{cap}^{ \textit{Mmax}}_{ \textit{m}}}{{\lvert}\mathcal{M}_{p}{\rvert}}}\cdot\frac{\sum_{m\in\mathcal{M}_{p}}\sum_{f} \textit{pay}^{ \textit{Macqu}}_{ \textit{mft}}}{{\lvert}\mathcal{M}_{p}{\rvert}\cdot{\lvert}\mathcal{F}{\rvert}}\cdot\frac{1}{T\cdot\overline{ \textit{AD}}_{p}}.$$

In the first term, we estimate the mean number of modules required to fulfill the mean demand for end product $p$. The second term describes the mean payments per unit for acquiring the modules for the production of end product $p$.

The mean payments $\overline{ \textit{pay}^{ \textit{Mhold}}_{ \textit{pt}}}$ for holding modules at a facility for each end product $p$ are calculated as follows:

24 $$\overline{ \textit{pay}^{ \textit{Mhold}}_{ \textit{pt}}}=\frac{\overline{ \textit{AD}}_{p}\cdot\frac{\sum_{m\in\mathcal{M}_{p}}\sum_{f}cf^{ \textit{Pprod}}_{ \textit{pmft}}}{{\lvert}\mathcal{M}_{p}{\rvert}\cdot{\lvert}\mathcal{F}{\rvert}}}{\frac{\sum_{m\in\mathcal{M}_{p}} \textit{cap}^{ \textit{Mmax}}_{ \textit{m}}}{{\lvert}\mathcal{M}_{p}{\rvert}}}\cdot\frac{\sum_{m\in\mathcal{M}_{p}}\sum_{f} \textit{pay}^{ \textit{Mhold}}_{ \textit{mft}}}{{\lvert}\mathcal{M}_{p}{\rvert}\cdot{\lvert}\mathcal{F}{\rvert}}\cdot\frac{1}{\overline{ \textit{AD}}_{p}}.$$

The estimated number of required facilities $\overline{ \textit{NumF}}$ is derived as follows:

25 $$\overline{ \textit{NumF}}=\frac{\sum_{p}\overline{ \textit{AD}}_{p}}{\sum_{f}\frac{(1+ \textit{cap}^{ \textit{Ffac}}_{f})\cdot\sum_{p}\overline{ \textit{AD}}_{ \textit{p}}\cdot(1+ \textit{VC}^{d})}{{\lvert}\mathcal{F}{\rvert}}}.$$

This estimation is used to determine the mean payments $\overline{ \textit{pay}^{ \textit{fac}}_{ \textit{pt}}}$ for establishing and running facilities to produce one unit of end product $p$ in period $t$.

26 $$\overline{ \textit{pay}^{ \textit{fac}}_{ \textit{pt}}}=\overline{ \textit{NumF}}\cdot\left(\frac{\sum_{f}\frac{ \textit{pay}^{ \textit{est}}_{ \textit{ft}}}{{\lvert}\mathcal{F}{\rvert}}}{T\cdot\sum_{ \textit{p'}}\overline{ \textit{AD}}_{ \textit{p'}}}+\frac{\sum_{f}\frac{ \textit{pay}^{ \textit{open}}_{ \textit{ft}}}{{\lvert}\mathcal{F}{\rvert}}}{\sum_{ \textit{p'}}\overline{ \textit{AD}}_{ \textit{p'}}}\right).$$

Furthermore, end-product-specific additional mark-ons $W^{P}_{p}\sim U[0.3,0.5]$ and retailer-specific additional mark-ons $W^{R}_{r}\sim U[0.05,0.1]$ are defined to determine (incoming) payments $\textit{pay}^{ \textit{sell}}_{ \textit{prt}}$ as follows:

$$\textit{pay}^{ \textit{Psell}}_{ \textit{prt}}=\left(1+W^{P}_{p}+W^{R}_{r}\right)\cdot$$

27 $$\begin{aligned}\qquad\left(\overline{ \textit{pay}^{ \textit{Cacqu}}_{ \textit{pt}}}+\overline{ \textit{pay}^{ \textit{Ctrans}}_{ \textit{pt}}}+\overline{ \textit{pay}^{ \textit{Pprod}}_{ \textit{pt}}}+\overline{ \textit{pay}^{ \textit{Ptrans}}_{ \textit{prt}}}+\overline{ \textit{pay}^{ \textit{Macqu}}_{ \textit{pt}}}+\overline{ \textit{pay}^{ \textit{Mhold}}_{ \textit{pt}}}+\overline{ \textit{pay}^{ \textit{fac}}_{ \textit{pt}}}\right).\end{aligned}$$
